# The molecular basis of the neutralization breadth of the RBD-specific antibody CoV11

**DOI:** 10.3389/fimmu.2023.1178355

**Published:** 2023-06-02

**Authors:** William D. Tolbert, Yaozong Chen, Lulu Sun, Mehdi Benlarbi, Shilei Ding, Rohini Manickam, Emily Pangaro, Dung N. Nguyen, Suneetha Gottumukkala, Marceline Côté, Frank J. Gonzalez, Andrés Finzi, Zahra R. Tehrani, Mohammad M. Sajadi, Marzena Pazgier

**Affiliations:** ^1^ Infectious Disease Division, Department of Medicine of Uniformed Services University of the Health Sciences, Bethesda, MD, United States; ^2^ Laboratory of Metabolism, Center for Cancer Research, National Cancer Institute, National Institutes of Health (NIH), Bethesda, MD, United States; ^3^ Centre de recherche du Centre hospitalier de l'Université de Montréal (CHUM), Montreal, QC, Canada; ^4^ Département de Microbiologie, Infectiologie et Immunologie, Université de Montréal, Montreal, QC, Canada; ^5^ Department of Biochemistry, Microbiology and Immunology, and Centre for Infection, Immunity, and Inflammation, University of Ottawa, Ottawa, ON, Canada; ^6^ Division of Vaccine Research, Institute of Human Virology, University of Maryland School of Medicine, Baltimore, MD, United States; ^7^ Division of Clinical Care and Research, Institute of Human Virology, University of Maryland School of Medicine, Baltimore, MD, United States; ^8^ Department of Medicine, Baltimore Veterans Health Administration (VA) Medical Center, Baltimore, MD, United States

**Keywords:** SARS-CoV-2, neutralizing antibody, VH 3-53 germline gene (IGHV3-53*01), receptor binding domain (RBD), variants of concern (VOC)

## Abstract

SARS-CoV-2, the virus behind the COVID-19 pandemic, has changed over time to the extent that the current virus is substantially different from what originally led to the pandemic in 2019–2020. Viral variants have modified the severity and transmissibility of the disease and continue do so. How much of this change is due to viral fitness versus a response to immune pressure is hard to define. One class of antibodies that continues to afford some level of protection from emerging variants are those that closely overlap the binding site for angiotensin-converting enzyme 2 (ACE2) on the receptor binding domain (RBD). Some members of this class that were identified early in the course of the pandemic arose from the V_H_ 3-53 germline gene (IGHV3-53*01) and had short heavy chain complementarity-determining region 3s (CDR H3s). Here, we describe the molecular basis of the SARS-CoV-2 RBD recognition by the anti-RBD monoclonal antibody CoV11 isolated early in the COVID-19 pandemic and show how its unique mode of binding the RBD determines its neutralization breadth. CoV11 utilizes a heavy chain V_H_ 3-53 and a light chain V_K_ 3-20 germline sequence to bind to the RBD. Two of CoV11’s four heavy chain changes from the V_H_ 3-53 germline sequence, $Thr_{FWR\;H1}^{28}$ to Ile and $Ser_{CDR\;H1}^{31}$ to Arg, and some unique features in its CDR H3 increase its affinity to the RBD, while the four light chain changes from the V_K_ 3-20 germline sequence sit outside of the RBD binding site. Antibodies of this type can retain significant affinity and neutralization potency against variants of concern (VOCs) that have diverged significantly from original virus lineage such as the prevalent omicron variant. We also discuss the mechanism by which V_H_ 3-53 encoded antibodies recognize spike antigen and show how minimal changes to their sequence, their choice of light chain, and their mode of binding influence their affinity and impact their neutralization breadth.

## 1 Introduction

Since the beginning of the COVID-19 pandemic, SARS-CoV-2 has been accumulating mutations to enhance infectivity and to avoid immune pressure (1). While SARS-CoV-2 has a much lower mutation rate than other RNA viruses such as influenza or HIV-1, it still mutates in response to its environment and over time due to genetic drift (2). Because SARS-CoV-2 has one major viral protein on its surface accessible to antibodies, the spike glycoprotein that is responsible for both target cell recognition and viral entry, it has become the focus of many of these mutations. The spike protein is a membrane-anchored trimer that is cleaved into S1 and S2 subunits by furin in the expressing cell (3, 4). S1 contains an N-terminal domain and a receptor binding domain (RBD) that recognizes the target cell receptor angiotensin-converting enzyme 2 (ACE2). S2 contains the fusion machinery. After the spike protein binds its target cell, it must be further proteolyzed to expose the fusion peptide in S2, which then inserts into the target cell membrane. This can occur at the cell surface of the target cell by proteases such as TMPRSS2 or in endosomes by proteases such as the cathepsins (5). Cell fusion and viral entry begin with removal of S1 from the trimer, which destabilizes the trimer and causes conformational rearrangements in S2. The initiating event in this process is the binding of the RBD to ACE2, which makes it a prime target for neutralizing antibodies (6–8).

The RBD sits at the top of the trimer and can exist in two different conformations, one with the RBD up and the ACE2 binding site accessible to the solvent and one with the RBD down and the ACE2 binding site occluded within the trimer (4). Each RBD in the trimer can exist in either conformation giving the spike a range of conformations from fully closed with all three RBDs in the down position to fully open with all three RBDs in the up conformation. One of the first mutations in the spike protein to spread and outcompete the original strain was the Asp^614^-to-Gly (D614G) mutation that removed a stabilizing hydrogen bond that increased the propensity for the RBD to be in the up conformation (9). This mutation likely increased infectivity by increasing the percentage of RBDs accessible to ACE2 for binding (10), but it came at a cost to the virus. It also made the RBD more accessible to neutralizing antibodies. Since then, viral variants such as alpha to the more recent flavors of omicron subvariants have appeared with mutations that can nullify the activity of some of these neutralizing antibodies. This has enabled the virus to continue to infect individuals even when they had been vaccinated or infected by a previous strain.

One group of antibodies that was identified earlier in the pandemic and continues to neutralize the virus and its emerging variants relatively well are those derived from the V_H_ 3-53 (IGHV3-53*01) heavy chain germline gene with short heavy chain complementarity-determining region 3s (CDR H3s) (11). These antibodies closely match the ACE2 binding site on the RBD, explaining in part their continued activity against the virus (12, 13). Initial reports also suggested that they could represent as much as 10% of the neutralizing antibody response elicited by SARS-CoV-2 (11). Structures of the first antibodies of this type were quickly determined and confirmed that they overlap the ACE2 binding site on the RBD (11–20). The receptor binding ridge on the RBD places a boundary on CDR H3 length and provides an explanation for the preference of short CDR H3s. Indeed when examples of V_H_ 3-53 encoded SARS-CoV-2 neutralizing antibodies with longer CDR H3s were determined, many were found to bind the RBD differently (12). The high frequency of this germline gene in the neutralization response to SARS-CoV-2 could be the result of two independent factors, the prevalence of the V_H_ 3-53 (and closely related V_H_ 3-66) germline genes in the general population, one study estimated a frequency of approximately 1% for V_H_ 3-53 in healthy individuals who had not been exposed to SARS-CoV-2 (21), and the low degree of somatic hypermutation necessary to confer specificity, as few as two mutations in the germline sequence was enough to increase one antibody’s affinity to the RBD from 407 nM to 3.6 nM (13).

Here, we report the structure of one example of a V_H_ 3-53 encoded SARS-CoV-2 neutralizing antibody, CoV11, and show how its mode of binding to the SARS-CoV-2 RBD leads to neutralization breadth. CoV11 was isolated from an infected individual early in the course of the pandemic but continues to neutralize many SARS-CoV-2 variants of concern (VOCs) relatively well. While V_H_ 3-53 encoded antibodies have similar sequences and modes of binding to the SARS-CoV-2 RBD, small differences in sequence and their choice of light chain influence their affinity and impact their neutralization breadth.

## 2 Materials and methods

### 2.1 Isolation of RBD-specific B cells and CoV11 antibody selection

CoV11 was isolated from a 76-year-old man with history of severe COVID-19, infected in February 2020 with high titer of neutralizing antibodies against the virus. CoV11 (also known as CoVIC-079) has been previously published (22). Briefly, memory B cells were isolated from PBMCs using the EasySep™ Human Memory B Cell Isolation Kit (STEMCELL Technologies). Cells were incubated with biotin-conjugated SARS-CoV-2 Spike antigen (LakePharma) and then, after washing, labeled by incubation with TotalSeq™-C0953 PE Streptavidin (BioLegend). The cell surface labeled single-cell suspension was loaded onto a 10x Genomics Chromium Controller microfluidics chip (10x Genomics) and a VDJ library was prepared based on the manufacturer’s instructions. A subset of cells containing the surface barcode were selected and their VDJ sequences were cloned into IgG1 heavy and light chain vectors. The recombinant plasmids were then co-transfected into FreeStyle-293 cells for expression and the secreted antibodies were purified from culture supernatants after incubations of 1 week by Protein A affinity chromatography. CoV11 was identified as one of the antibodies that strongly neutralized the virus.

### 2.2 Protein production and purification

SARS-CoV-2 RBD was cloned from an expression plasmid of the SARS-CoV-2 2P soluble spike protein (a gift from Jason McLellan) (residues 319–537) into an expression plasmid with an N-terminal leader sequence and a C-terminal 6-histidine tag. SARS-CoV-2 RDB_delta_ was made by adding T478K and L452R mutations to the wild-type RBD using a QuikChange mutagenesis kit (Stratagene). CoV11 IgG was produced by transection of CoV11 heavy and light chain plasmids into expi293F cells and SARS-CoV-2 RBD_wt_ or RDB_delta_ were produced by transfection into GnT1^-^ 293F cells. Cells were then grown in suspension for an additional 7 days at 37°C and 90% humidity. Cells were pelleted by centrifugation and the medium was filtered through a 0.2-micron filter. CoV11 IgG was purified from medium passed over a HiTrap protein A column (Cytiva) equilibrated with phosphate buffered saline (PBS), pH 7.2. The column was washed with PBS and the IgG was eluted with 0.1 M glycine, pH 3.0. Eluted fractions were immediately diluted 10:1 with 1 M tris(hydroxymethyl)aminomethane-HCl (Tris-HCl), pH 8.5. Eluted protein was concentrated to approximately 10 mg/ml and the buffer was exchanged for PBS, pH 7.2. SARS-CoV-2 RBD was purified using a HisTrap column (Cytiva) equilibrated in wash buffer, 25 mM Tris-HCl, pH 8.0, and 500 mM sodium chloride. Medium was passed over the column and the column was washed with wash buffer. Protein was eluted with 25 mM Tris-HCl and 500 mM imidazole, pH 8.0. Eluted protein was concentrated, and the sample was loaded onto a Superdex 200 gel filtration column (Cytiva) equilibrated in 10 mM Tris-HCl, pH 7.2, and 100 mM ammonium acetate. Fractions corresponding to the correct RBD size were concentrated and used for complex formation.

CoV11 Fab was generated from IgG by papain cleavage. IgG in PBS was first exchanged to Fab digest buffer, 10 mM sodium phosphate, pH 7.0, and 5 mM cysteine. IgG (10 mg) at approximately 10 mg/ml was added to 3 ml of papain-linked agarose slurry (Thermo Fisher) previously equilibrated in Fab digest buffer. The digest was incubated at 37°C for 3 h and then filtered to remove the papain. Filtered protein was passed over a protein A column equilibrated in PBS, pH 7.2. Flow through fractions containing the Fab were concentrated and then loaded onto a Superdex 200 gel filtration column (Cytiva) equilibrated in 10 mM Tris-HCl, pH 7.2, and 100 mM ammonium acetate. The Fab elution peak corresponding to a molecular weight of approximately 50 kDa was collected and concentrated for use in complex formation or SPR.

Complexes were made by mixing Fab and RBD at a 1:1 ratio and incubation on ice for 30 min. The sample was then loaded onto a Superdex 200 gel filtration column (Cytiva) equilibrated in 10 mM Tris-HCl, pH 7.2, and 100 mM ammonium acetate. Fractions corresponding to the complex molecular weight were collected and concentrated to approximately 10 mg/ml for use in crystallization trials.

### 2.3 SARS-CoV-2 pseudovirus production

SARS-CoV-2 S-pseudotyped virus-like particles (VLPs) containing the synthetic firefly luciferase (Luc2) reporter were prepared using the BEI NR-52948 kit as described in (22). To generate VLP pseudotyped with spikes of different SARS-CoV-2 VOCs, spike pseudotyping vector plasmids, including WA-1/2020 (WT), D614G (BEI Resources), gamma (a gift from Dr. Robert Petrovich from NIEHS), alpha, beta, epsilon, iota, delta (InvivoGen), and omicron BA.1 (Sino Biological), BQ.1.1 (23, 24) and XBB.1.5 (25) VOCs, were used. Sixteen to 24 h post seeding, 293T cells (Thermo Fisher Scientific) were co-transfected with respective spike plasmid or VSV G (positive control), lentiviral backbone, and three helper plasmids encoding Gag, Tat1b, and Rev1b (BEI Resources). At 72 h post-transfection, the supernatant was harvested and clarified by a 0.45-μm filter. To determine viral titers, hACE2-expressing 293T cells (gift from Dr. Allison Malloy, USUHS) were infected with serial VLP dilutions. Forty-eight to 60 h post-infection, luciferase signal was detected by the Bright-Go Luciferase Assay System (Promega) for titer estimations (26). VLPs were concentrated by a homemade fourfold lentivirus concentrator (protocol of MD Anderson) and stored at 4°C for short-term use or −20°C for longer storage.

### 2.4 *In vitro* neutralization assay

For *in vitro* neutralization assays, 50-μl serial dilutions of CoV11 or synagis with final concentrations from 0.005 to 50 ng/μl were pre-incubated with 50 μl of SARS-CoV-2 spike-pseudotyped VLPs (~106 RLU/ml) of WT or one of eight VOCs in 96-well plates at 37°C for 1 **h**. Subsequently, Hace2-expressing 293T cells (1.25 × 10^4^ cells/well) in 50 μl of culture medium were added and incubated at 37°C for 48 **h** before luciferase signal measurement with the Bright-Go Luciferase Assay System (Promega). Data analysis and normalization followed the protocol described previously in (26). Pseudoviral neutralization experiments presented in **Figure S3** were performed as we previously reported (23). Briefly, 293T cells were transfected by the calcium phosphate method with the lentiviral vector Pnl4.3 R-E- Luc (NIH AIDS Reagent Program) and a plasmid encoding for SARS-CoV-2 Spikes at a ratio of 5:4. Two days post-transfection, cell supernatants were harvested and stored at –80°C until use. 293T-ACE2 target cells were seeded at a density of 1×10^4^ cells/well in 96-well luminometer-compatible tissue culture plates (Perkin Elmer) 24 **h** before infection. Recombinant viruses in a final volume of 100 μl were incubated with the indicated concentrations of mAbs for 1 **h** at 37°C and were then added to the target cells followed by incubation for 48 **h** at 37°C; cells were lysed by the addition of 30 μl of passive lysis buffer (Promega) followed by one freeze–thaw cycle. An LB941 TriStar luminometer (Berthold Technologies) was used to measure the luciferase activity of each well after the addition of 100 μl of luciferin buffer (15 Mm MgSO_4_, 15 Mm KPO_4_ [Ph 7.8], 1 Mm ATP, and 1 Mm dithiothreitol) and 50 μl of 1 Mm d-luciferin potassium salt (Prolume). Relative lighting unit (RLU) for luciferase activity was recorded and the ratio to “no mAb” was calculated. VSV-G pseudoviral particles were used as a specificity control. Surface plasmon resonance (SPR) measurements were carried out as described in (27). All assays were performed on a Biacore 3000 (Cytiva) at room temperature using 10 Mm HEPES, Ph 7.5, 150 Mm NaCl and 0.05% Tween 20 as running buffer. For the kinetic measurement, ~80–200 RU of CoV11 IgG was immobilized on a Protein A sensor chip (Cytiva) and twofold serial dilutions of SARS-CoV-2 RBD from WT or one of six VOCs was then injected as solute analyte with concentrations ranging from 6.25 to 200 Nm. For all kinetic assays, the sensor chip was regenerated using 10 Mm glycine, Ph 2.5, before the next cycle. Sensorgrams were corrected by subtraction of the corresponding blank channel as well as for the buffer background and kinetic constants were determined using a 1:1 Langmuir model with the BIAevaluation software (Cytiva) as shown in **Table S2** and **Figure S1**. Goodness of fit of the curve was evaluated by the χ^2^ of the fit with a value below 3 considered acceptable.

### 2.5 Bio-layer interferometry

Binding kinetics were performed using an Octet RED96e system (ForteBio) with shaking at 1,000 RPM. Protein A (ProA) biosensors were hydrated in water prior to use. CoV11 mAb was loaded into ProA biosensors at 12.5 µg/ml in 10X kinetic buffer (Fortebio) for 120 s. Loaded biosensors were placed in 10X kinetic buffer (ForteBio) for 120 s for baseline equilibration. Association of CoV11 mAb (in 10X kinetics buffer) to the different RBD proteins was carried out for 180 s at various RBD concentrations in a twofold dilution series from 200 nM to 6.25 nM, prior to dissociation for 300 s. The data were baseline subtracted prior to fitting performed using a 1:1 binding model and the ForteBio data analysis software. Calculation of on rates (*k*_a_), off rates (*k*_d_), and affinity constants (*K*_D_) were computed using a global fit applied to all data.

### 2.6 Crystallization and data collection

Initial crystals were grown from Molecular Dimensions sparse matrix screens, specifically ProPlex Eco and MacroSol Eco screens. Crystals were then reproduced and optimized using the hanging-drop, vapor diffusion method. CoV11 Fab-RBD_wt_ crystals were grown from 15% polyethylene glycol (PEG) 4000, 150 mM ammonium sulfate, and 100 mM 2-(N-morpholino)ethanesulfonic acid (MES), pH 6.0. CoV11 Fab-RBD_delta_ crystals were grown from 10% PEG 4000, 200 mM sodium acetate, and 100 mM sodium citrate, pH 5.5 (crystal form 1), and 12% PEG 8000 and 100 mM sodium phosphate, pH 6.5 (crystal form 2). Prior to freezing, crystals were briefly soaked in the crystallization condition supplemented with 20% of 2-methyl-2,4-pentanediol (MPD) as cryoprotectant.

Diffraction data were collected at the Stanford Synchrotron Radiation Light Source (SSRL) beamline 12-2 on a Dectris Pilatus 6M area detector. All data were processed and reduced with HKL3000 (28). Structures were solved by molecular replacement with PHASER from the CCP4 suite (29) based on the coordinates from PDB ID 7JMP. Refinement was carried out with Refmac5 (29) and/or Phenix (30) and model building was done with COOT (29). Data collection and refinement statistics are shown in **Table 1**. Ramachandran statistics were calculated with MolProbity and illustrations were prepared with Pymol Molecular graphics (http://pymol.org).

**Table 1:** Combined kinetic constants (BLI and SPR) of CoV11 binding to the RBD of SARS-CoV-2 wt and selected VOCs.

Immobilized ligand	Flow analyte	*k*_a_ (M^−1^ s^−1^)	*k*_d_ (s^−1^)	*K*_D_ (nM)	*K*_D_ fold increase as compared to RBD_wt_
CoV11 IgG	RBD_wt_	8.2 × 10^4^ ± 2.6 × 10^4^	1.7 × 10^−4^ ± 4.7 × 10^−5^	2.1 ± 0.4	1
	RBD_alpha_	5.9 × 10^4^ ± 2.7 × 10^4^	v6.8 × 10^−4^ ± 1.1 × 10^−3^	2.3 ± 0.9	1.1
	RBD_beta_	2.7 × 10^4^ ± 3.5 × 10^5^	1.1 × 10^−2^ ± 4.8 × 10^−3^	138 ± 124	66
	RBD_epsilon_	1.1 × 10^5^ ± 6.7 × 10^4^	3.4 × 10^−4^ ± 3.9 × 10^−4^	2.5 ± 1.6	1.2
	RBD_iota_	1.3 × 10^5^ ± 8.3 × 10^4^	5.1 × 10^−4^ ± 4.6 × 10^−4^	3.7 ± 0.8	1.8
	RBD_delta_	1.1 × 10^5^ ± 6.5 × 10^4^	3.1 × 10^−4^ ± 2.8 × 10^−4^	2.5 ± 0.8	1.2
	RBD_omicron BA.2_	1.2 × 10^5^ ± 9.3 × 10^4^	7.4 × 10^−3^ ± 1.1 × 10^−3^	85 ± 45	40
	RBD_omicron BQ.1.1_	N.D.	N.D.	N.D.	–
	RBD_omicron XBB.1.5_	N.D.	N.D.	N.D.	–

The equilibrium dissociation constants (K_D_), association constants (k_a_), and dissociation constants (k_d_) are as shown. K_D_ values were determined using a 1:1 Langmuir model. Values are the average of three or four experiments with standard deviations as shown. N.D., not detected.

## 3 Results

### 3.1 CoV11 potently neutralizes SARS-CoV-2 wild type and retains neutralization activity against emerging variants including delta and omicron

**Figure 1:**
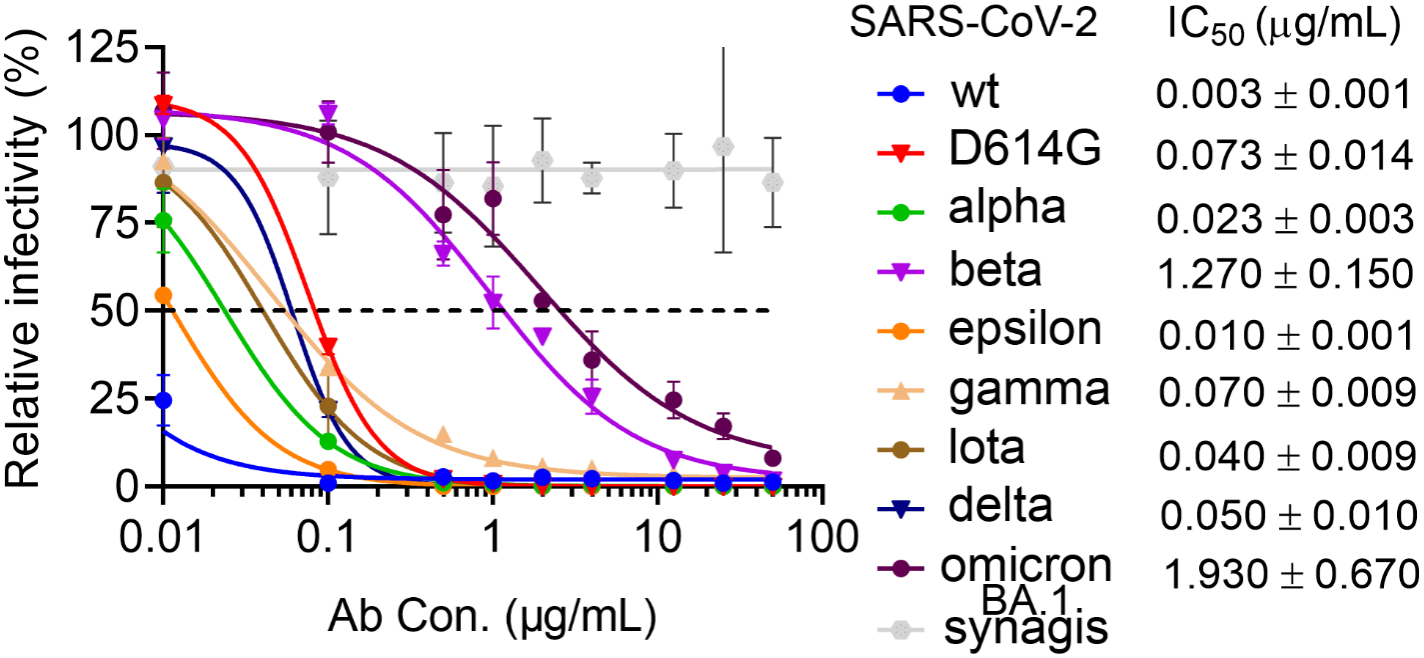
Neutralization activity of COV11 against SARS-CoV-2_wt_ and VOCs. Dose–response neutralization curves of SARS-CoV-2 pseudotyped lentivirus with eight SAR2-CoV-2 S variants. hACE2 expressing 293T cells were infected with different variants of SARS-CoV-2 PsV in the presence of CoV11, Synagis IgG (negative control), or PBS saline. Infectivity was quantified by the cellular luciferase signal 48 h post-infection. Relative infectivity was normalized by the luciferase signal in infected cells without intervention (PBS saline). EC_50_ values for individual strains are shown to the right.

CoV11 is derived from the heavy chain V_H_ 3-53 (IGHV3-53*01) and light chain V_K_ 3-20 (IGKV3-20*01) germline genes and has four mutations in its heavy chain and four in its light chain relative to germline sequences. It also has a short CDR H3 of 10 amino acids, which is the product of heavy chain joining and diversity germline sequences. When tested for recognition of RBD of different SARS-CoV2 variants, CoV11 binds the original, wild type (wt), SARS-CoV-2 strain RBD with a *K*_D_ of 2.1 nM and potently neutralizes SARS-CoV-2 wt pseudotyped viruses with an IC_50_ of 0.003 µg/ml (**Tables 1**, **S2**, **S3** and **Figure 1**). CoV11’s affinity to the RBD and neutralization potency are reduced by SARS-CoV-2 VOCs, but to different degrees, most likely due to the avidity of an IgG as compared to a Fab. CoV11’s affinity to alpha (B.1.1.7) RBD is 2.3 nM and that to beta (B.1.351) RBD is 10.4 nM as determined by SPR (**Table S2**) and 202 nM as determined by bio-layer interferometry (BLI) (**Table S3**); CoV11’s affinity to epsilon (B.1.427/B.1.429) RBD is 2.5 nM, that to iota (B.1.526) RBD is 3.7 nM, that to delta (B.1.617.2) RBD is 2.5 nM, and that to omicron BA.2 RBD is 24.4 nM as determined by SPR and 105 nM as determined by BLI. We observed higher K_D_s for beta and omicron BA.2 determinations by BLI as compared to those determined by SPR. This could be in part be due to differences in the two techniques, but for the beta RBD, it could also be due to the use of commercially prepared protein used for BLI measurements versus in-house-produced protein used for SPR measurements. The K_D_s for the other RBDs were comparable and are averaged (**Table 1**). In comparison, the IC_50_ of CoV11 IgG to pseudotyped alpha is 0.02 µg/ml; beta, 1.3 µg/ml; epsilon, 0.01 µg/ml; gamma (P.1), 0.07 µg/ml; iota, 0.04 µg/ml; delta, 0.05 µg/ml; omicron (B.1.1.529.1 or BA.1), 1.9 µg/ml (**Figure 1**). Thus, the avidity of an IgG relative to a Fab largely mitigates small losses in CoV11’s affinity due to escape mutations at the antibody–RBD interface in VOCs. Of note, the most detrimental mutations in VOCs for CoV11 occur in the beta and omicron RBDs (**Figure 1** and **Tables 1**, **S2**, and **S3**).

### 2.2 CoV11 recognizes the RBD by largely overlapping the ACE2 binding site, making it a member of the class 1 of SARS-CoV-2 neutralizing antibodies

**Figure 2:**
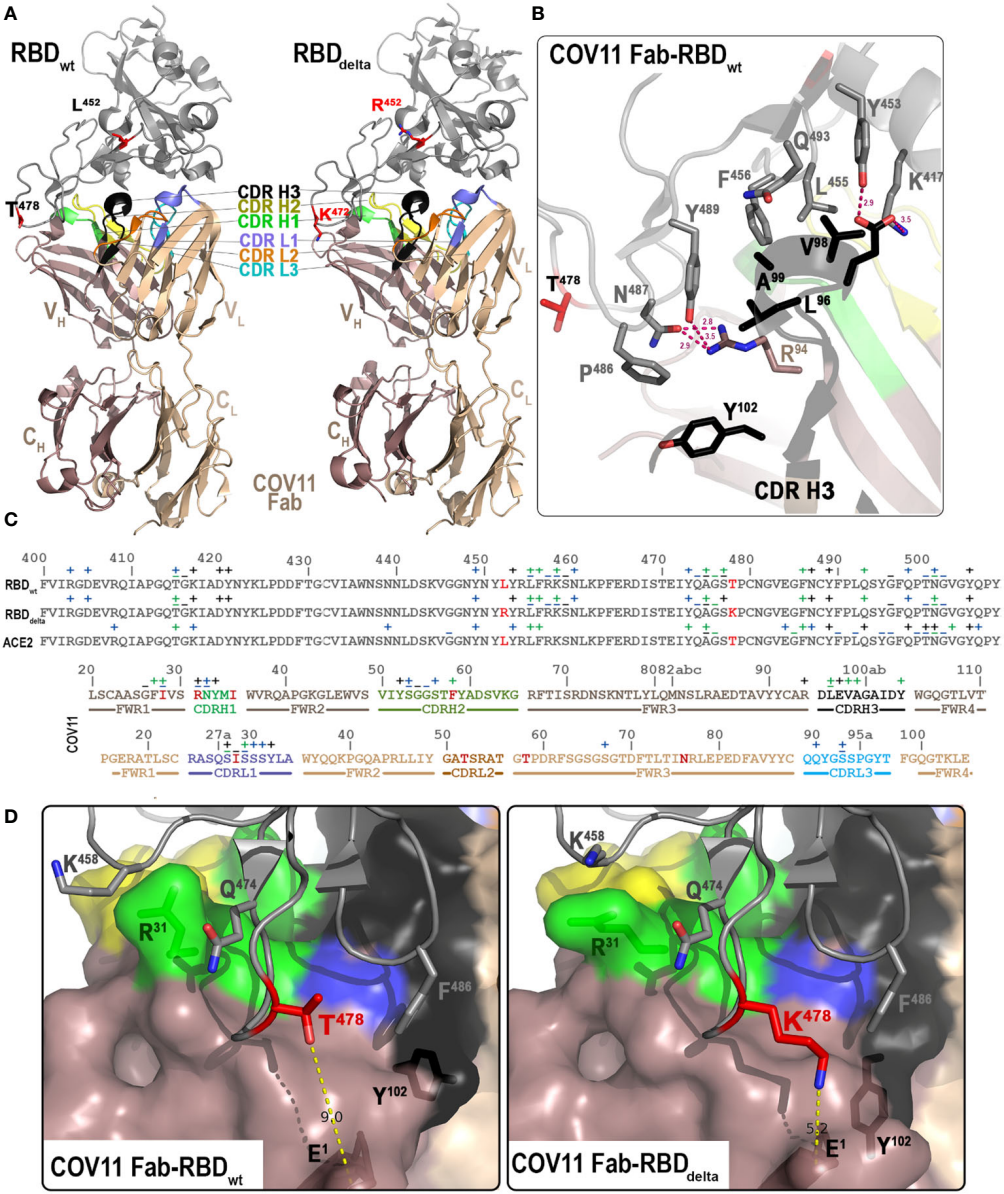
Crystal structures of the CoV11 Fab-RBD complexes. **(A)** Overall structure of the complex of CoV11 Fab-RBD_wt_ (left) and CoV11 Fab-RBD_delta_ (right) are shown as a ribbon diagram. The complementarity-determining regions (CDRs) of CoV11 Fab are colored as follows: CDR H1 is green, CDR H2 is yellow, CDR H3 is black, CDR L1 is light blue, CDR L2 is orange, and CDR L3 is cyan. Residues T/K ^478^ and L/R^452^ mutated in delta as compared to wt are shown as sticks and colored red. **(B)** Close-up view into the interaction network mediated by the CoV11 CDR H3. Complex interface residues are shown as sticks and H-bonds are shown as magenta dashed lines. **(C)** Binding footprints of CoV11 and ACE2 on the RBD and the RBD on the CoV11 heavy and light chains. Contact residues defined by a 5-Å cutoff are marked above the sequence with (+) for side chain and (−) for main chain to indicate the type of contact. Contact types are colored as follows: hydrophilic (blue), hydrophobic (green), and both (black). Residues that differ from the V_H_ 3-53 or V_K_ 3-20 germline sequences on the heavy and light chains respectively are colored red. CDRs are colored as in **(A, D)** Interaction network around T/K ^478^. CoV11 and RBD residues in which the conformation differs between CoV11 Fab-RBD_wt_ (left) and CoV11 Fab-RBD_delta_ (right) complexes are shown as sticks. The distance between T/K ^478^ of the RBD and N-terminal E^1^ of CoV11 heavy chain are shown with yellow dashed lines. A molecular surface is displayed over the Fab and the RBD is shown as a ribbon diagram.

To get a better understanding of how CoV11 recognizes SARS-CoV2 spike, we determined the crystal structure of CoV11 Fab in complex with the wt and the delta (B.1.617.2) variants of the SARS-CoV-2 RBD. CoV11 Fab-RBD_wt_ crystals belonged to space group P2_1_2_1_2_1_ and diffracted to 2.05 Å with one CoV11 Fab-RBD_wt_ complex in the asymmetric unit (**Figure 2A** and **Table 2**). The wt RBD complex was refined to an *R*/*R*_free_ of 0.166/0.198. CoV11 Fab-RBD_delta_ crystallized in two different space groups, P2_1_2_1_2_1_, which diffracted to 2.05 Å, and C2, which diffracted to 2.4 Å. Both crystals had one CoV11 Fab-RBD_delta_ complex in the asymmetric unit. The orthorhombic delta RBD P2_1_2_1_2_1_ crystal form was refined to an *R*/*R*_free_ of 0.177/0.208 and the monoclinic delta RBD C2 crystal form was refined to an *R*/*R*_free_ of 0.179/0.224. Complete data collection and refinement statistics can be found in **Table 2**.

**Table 2:** Data collection and refinement statistics.

	CoV11 Fab-SARS-CoV-2 RBD	CoV11 Fab-SARS-CoV-2 delta RBD (1)	CoV11 Fab-SARS-CoV-2 delta RBD (2)
Data collection			
Wavelength, Å	0.979	0.979	0.979
Space group	P2_1_2_1_2_1_	P2_1_2_1_2_1_	C2
Cell parameters			
*a*, *b*, *c*, Å	55.1, 111.3, 142.9	85.8, 103.6, 112.1	194.5, 86.6, 57.7
α, β, γ, °	90, 90, 90	90, 90, 90	90, 99.9, 90
Molecules/a.u.	3	3	3
Resolution, (Å)	50–2.04 (2.08–2.04)	50–2.05 (2.09–2.05)	50–2.4 (2.44–2.4)
# of reflections			
Total	219,416	334,796	102,672
Unique	54,854	59,785	34,224
$R_{merge}^{a}$ , %	14.1 (63.7)	12.8 (82.7)	12.6 (38.0)
$R_{pim}^{b}$ , %	7.3 (42.5)	5.6 (46.7)	8.1 (29.4)
*CC*_1/2_ ^c^	0.98 (0.57)	0.99 (0.49)	0.97 (0.80)
*I*/σ	11.6 (1.65)	19.9 (1.3)	17.5 (1.9)
Completeness, %	98.0 (92.1)	93.3 (74.8)	92.3 (72.0)
Redundancy	4.0 (2.6)	5.6 (3.4)	3.0 (1.9)
Refinement Statistics			
Resolution, Å	50.0–2.05	50.0–2.05	50.0–2.4
*R* ^d^ %	16.6	17.7	17.9
$R_{free}^{e}$ , %	19.8	20.8	22.4
# of atoms			
Protein	4,857	4,825	4,746
Water	567	243	85
Ligand/Ion	55	27	40
Overall *B* value (Å)^2^			
Protein	33	51	60
Water	40	50	50
Ligand/Ion	75	94	107
RMSD^f^			
Bond lengths, Å	0.008	0.008	0.010
Bond angles, °	0.98	1.0	1.2
Ramachandran^g^			
Favored, %	95.1	96.6	94.7
Allowed, %	3.9	3.2	4.6
Outliers, %	1.0	0.2	0.7
PDB ID	7S4S	7URQ	7URS

Values in parentheses are for the highest-resolution shell.

a R_merge_ = ∑│I −<I>│/∑I, where I is the observed intensity and<I> is the average intensity obtained from multiple observations of symmetry-related reflections after rejections.

b R_pim_ = as defined in (31).

c CC_1/2_ = as defined by Karplus and Diederichs (32).

d R = ∑║F_o_│ − │F_c_║/∑│F_o_│, where F_o_ and F_c_ are the observed and calculated structure factors, respectively.

e R_free_ = as defined by Brünger (33).

f RMSD = Root mean square deviation.

g Calculated with MolProbity.

CoV11 binds the RBD in what has become the characteristic mode of binding for V_H_ 3-53 encoded SARS-CoV-2 neutralizing antibodies by closely overlapping the ACE2 binding site on the RBD (**Figure 2A**). Binding to the RBD is achieved mainly through CDRs H1, H2, H3, and L1 [19.7%, 21.0%, 20.3%, and 19.9% of the total buried surface area (BSA) of CoV11, respectively] with small contributions from framework residues in the heavy chain (12.4%) and CDR L3 (4.9%); light chain framework residues (1.5%) and CDR L2 (0.3%) make almost no contribution to the total CoV11 BSA (**Table S1**). The bulk of these residues are encoded by the two CoV11 germline genes (IGHV3-53*01 and IGKV3-20*01), i.e., residues outside of the CDR H3, and account for the germline preference associated with this mode of binding to the RBD.

Most of the heavy chain framework residues that contribute to binding occur in framework region 1 preceding CDR H1 ( $Gly_{FWR\;H1}^{26}$ , $Phe_{FWR\;H1}^{27}$ , and $Ile_{FWR\;H1}^{28}$ —for clarity, antibody residues are referenced using Kabat numbering (34) in superscript and region in subscript and RBD and ACE2 residues are referenced using the residue number in superscript and the protein in subscript unless implicitly implied in the text) (**Figures 2B, C**). The carboxyl of $Gly_{FWR\;H1}^{26}$ makes a main chain hydrogen bond to the nitrogen of RBD residue $Ser_{RBDwt}^{27}$ and the side chains of $Phe_{FWR\;H1}^{27}$ and $Ile_{FWR\;H1}^{28}$ make hydrophobic van der Waals contacts to RDB residues $Ala_{RBDwt}^{475}$ and $Gly_{RBDwt}^{476}$ . $Phe_{FWR\;H1}^{27}$ also makes van der Waals contacts with $Phe_{RBDwt}^{486}$ .

CDR H1 residues $Arg_{CDR\;H1}^{31}$ , $Asn_{CDR\;H1}^{32}$ , and $Tyr_{CDR\;H1}^{33}$ increase the number of contacts to RBD residues in this region. $Arg_{CDR\;H1}^{31}$ makes a main chain hydrogen bond to RBD residue $Tyr_{RBDwt}^{473}$ and a side chain-mediated hydrogen bond to $G\ln_{RBDwt}^{474}$ in addition to hydrophobic contacts with $Ala_{RBDwt}^{475}$ and $Lys_{RBDwt}^{458}$ . $Asn_{CDR\;H1}^{32}$ makes a side chain-mediated hydrogen bond to the main chain carbonyl of $Ala_{RBDwt}^{475}$ , and the side chain of $Tyr_{CDR\;H1}^{33}$ makes a hydrogen bond to the main chain carbonyl of $Leu_{RBDwt}^{455}$ (**Figures 2B,C**).

CDR H2 contributions to the interface are mainly through the side chains of $Ser_{CDR\;H2}^{53}$ , $Ser_{CDR\;H2}^{56}$ , and $Phe_{CDR\;H2}^{58}$ and the main chains of $Gly_{CDR\;H2}^{54}$ and $Gly_{CDR\;H2}^{55}$ . $Ser_{CDR\;H2}^{53}$ makes a strong hydrogen bond to $Arg_{RBDwt}^{457}$ and weaker hydrogen bonds to $Tyr_{RBDwt}^{421}$ and $Tyr_{RBDwt}^{473}$ . $Ser_{CDR\;H2}^{56}$ makes a strong hydrogen bond to $Asp_{RBDwt}^{420}$ . The main chain nitrogen of $Gly_{CDR\;H2}^{54}$ makes a hydrogen bond to $Tyr_{RBDwt}^{421}$ while the main chain carbonyl makes a water-mediated hydrogen bond to the main chain of $Asn_{RBDwt}^{460}$ and the main chain nitrogen of $Gly_{CDR\;H2}^{55}$ makes water-mediated hydrogen bonds with the side chains of $Tyr_{RBDwt}^{421}$ and $Asn_{RBDwt}^{460}$ . $Phe_{CDR\;H2}^{58}$ makes side chain-mediated van der Waals contacts with $Thr_{RBDwt}^{415}$ and $Gly_{RBDwt}^{416}$ (**Figure 2C**).

CoV11’s CDR H3 interaction with the RBD begins with the arginine preceding the CDR H3, $Arg_{FWR\;H3}^{94}$ , which makes hydrogen bonds to RBD residues $Asn_{RBDwt}^{487}$ and $Tyr_{RBDwt}^{498}$ and van der Waals contacts with $Phe_{RBDwt}^{486}$ (**Figure 2B**). CDR H3 $Leu_{CDR\;H3}^{96}$ packs against $Leu_{RBDwt}^{455}$ , $Phe_{RBDwt}^{456}$ , and $Tyr_{RBDwt}^{489}$ , while $Glu_{CDR\;H3}^{97}$ makes hydrogen bonds to $Tyr_{RBDwt}^{453}$ and $Lys_{RBDwt}^{417}$ . $Val_{CDR\;H3}^{98}$ makes van der Waals contacts with $Tyr_{RBDwt}^{453}$ , $Leu_{RBDwt}^{455}$ , and $G\ln_{RBDwt}^{493}$ , and $Ala_{CDR\;H3}^{99}$ makes contacts with $Leu_{RBDwt}^{455}$ , $Phe_{RBDwt}^{456}$ , $Tyr_{RBDwt}^{489}$ , and $G\ln_{RBDwt}^{493}$ . Finally, $Tyr_{CDR\;H3}^{102}$ at the end of the CDR H3 packs against $Phe_{RBDwt}^{489}$ . Residues from the center of the CDR H3 do not interact with the RBD. Aside from $Arg_{FWR\;H3}^{94}$ and $Glu_{CDR\;H3}^{97}$ , most of the CDR H3 interface interactions are hydrophobic (**Figure 2C**).

The bulk of CoV11’s interactions from the light chain reside in CDR L1 (**Figure 2C**). $Ser_{CDR\;L1}^{27A}$ forms a hydrogen bond with $Gly_{RBDwt}^{502}$ and van der Waals contacts with $Asn_{RBDwt}^{501}$ and $Tyr_{RBDwt}^{505}$ . The main chains of $Ile_{CDR\;L1}^{28}$ and $Ser_{CDR\;L1}^{29}$ are mainly involved in hydrophobic contacts with $Tyr_{RBDwt}^{505}$ although $Ser_{CDR\;L1}^{29}$ also forms water-mediated hydrogen bonds with $Gly_{RBDwt}^{496}$ , $G\ln_{RBDwt}^{498}$ , and $Asn_{RBDwt}^{501}$ . The remaining contacts in CDR L1 are hydrophilic. $Ser_{CDR\;L1}^{30}$ forms a water-mediated hydrogen bond with $G\ln_{RBDwt}^{498}$ , $Ser_{CDR\;L1}^{31}$ forms a weak hydrogen bond with $Tyr_{RBDwt}^{449}$ , and $Tyr_{CDR\;L1}^{32}$ forms a hydrogen bond with $Tyr_{RBDwt}^{453}$ .

CDR L2 does not make any contacts with the RBD, but $Ser_{FWR\;L3}^{67}$ in the framework region preceding CDR L3 makes a weak hydrogen bond to $G\ln_{RBDwt}^{498}$ . All other interface residues reside in CDR L3. The carboxyl of $Gly_{CDR\;L3}^{92}$ forms a strong hydrogen bond with $Arg_{RBDwt}^{403}$ , the side chain of $Ser_{CDR\;L3}^{93}$ makes a hydrogen bond to $Tyr_{RBDwt}^{505}$ and a water-mediated hydrogen bond to $Asp_{RBDwt}^{405}$ , and $G\ln_{CDR\;L3}^{90}$ at the beginning of the CDR L3 also makes a weak hydrogen bond to $Tyr_{RBDwt}^{505}$ (**Figure 2C**).

### 2.3 CoV11 binds to the delta RBD with almost identical affinity to the wt RBD

The delta SARS-CoV-2 variant (B.1.617.2), which was originally identified in India in late 2020, quickly became the most prevalent SARS-CoV-2 variant in the US and the world in 2021 (35, 36). CoV11 binds to the wild-type RBD with an affinity of 2.1 nM and to the delta RBD with an affinity of 2.5 nM largely due to a slight increase in the *k*_off_ (*k*_d_) for the delta RBD (**Table 1**); the *k*_on_ (*k*_a_) for both RBDs is roughly equivalent. Delta contained two mutations in the RBD, Leu^452^ to Arg and Thr^478^ to Lys. The CoV11 Fab-RBD_delta_ complex shows that although these mutations sit outside of CoV11’s epitope footprint, they do make small changes to the way CoV11 binds to the RBD. The most notable difference is in how the N-terminus of the Fab sits relative to position 478 in the RBD (**Figures 2D, E**). The glutamic acid at the N-terminus of CoV11 is approximately 9 Å from $Thr_{RBDwt}^{478}$ but 5 Å from $Lys_{RBDdelta}^{478}$ . This is only partly due to the longer side chain on $Lys_{RBDdelta}^{478}$ . The change in the position of CoV11’s N-terminus also influences how $Tyr_{CDR\;H3}^{102}$ interacts with $Phe_{RBD}^{486}$ . In the wt RBD, $Tyr_{CDR\;H3}^{102}$ and $Phe_{RBD}^{486}$ display aromatic π stacking interactions, but in the delta RBD, the closer N-terminus shifts $Tyr_{CDR\;H3}^{102}$ such that it cannot interact with Phe^486^. The interaction between $Glu_{FWR\;H1}^{1}-Lys_{RBDdelta}^{478}$ may partially compensate for this loss. The closer N-terminus also causes some downstream changes to the interface. $Arg_{CDR\;H1}^{31}$ forms a hydrogen bond to the main chain of $G\ln_{RBD}^{474}$ in the wt RBD but makes stronger van der Waals interactions with the aliphatic portion of the $Lys_{RBDdelta}^{458}$ side chain in the delta RBD. $Ile_{CDR\;L1}^{28}$ is also closer to $Asn_{RBD}^{501}$ in the wt RBD, while $Ser_{CDR\;L1}^{29}$ is closer to Gln^498^ in the delta RBD. Most other differences are minor, which is mirrored by the less than twofold reduction in binding affinity for the delta RBD as compared to wt.

### 2.4 Some, but not all of CoV11’s changes relative to germline sequences increase CoV11’s affinity to the RBD

There are four sequence changes in the heavy chain of CoV11 relative to the V_H_ 3-53 germline sequence. In the first sequence change, the V_H_ 3-53 germline sequence has Thr at position 28, while CoV11 has Ile (**Figure S2**). This $Thr_{FWR\;H1}^{28}$ -to-Ile change in CoV11 increases van der Waals contacts with $Ala_{RBDwt}^{475}$ and $Gly_{RBDwt}^{476}$ . The second sequence difference, a germline $Ser_{CDR\;H1}^{31}$ -to-Arg change in CDR H1 adds one hydrogen bond to the CoV11–RBD interface; the side chain of $Ser_{CDR\;H1}^{31}$ is too short to make a similar hydrogen bond (**Figure 2C**). The third sequence difference, a $Ser_{CDR\;H1}^{35}$ -to-Ile change in CoV11, is not involved in the CoV11–RBD interface and likely has little if any contribution to CoV11 affinity for the RBD. Finally, the fourth sequence change resides in CDR H2. A $Tyr_{CDR\;H2}^{58}$ -to-Phe change in CoV11 relative to the germline sequence does not also seem to change the interface much except in that it switches the interface to use only the hydrophobic van der Waals interactions of $Phe_{CDR\;H2}^{58}$ instead of the both hydrophilic and hydrophobic interactions of $Tyr_{CDR\;H2}^{58}$ . Thus, the increases to RBD affinity for CoV11 over the germline gene largely come from two changes in sequence, $Thr_{FWR\;H1}^{28}$ to Ile and $Ser_{CDR\;H1}^{31}$ to Arg.

Sequence changes from the germline gene in the light chain contribute even less to the interface. CoV11 utilizes a light chain with four changes in sequence relative to the V_K_ 3-20 germline gene (**Figure S2**). The only residue that differs from the V_K_ 3-20 germline sequence involved in the CoV11–RBD interface is in CDR L1 ( $Val_{CDR\;L1}^{28}$ to Ile) (**Figure 2C**). $Ile_{CDR\;L1}^{28}$ , however, only interacts with the RBD *via* main chain residues, which suggests that the Val-to-Ile change makes little if any contribution to CoV11’s binding affinity. The three other changes in sequence relative to the V_K_ 3-20 germline ( $Ser_{CDR\;L2}^{52}$ to Thr, $Ile_{FWR\;L3}^{58}$ to Thr, and $Ser_{FWR\;L3}^{76}$ to Asn) sit well outside the CoV11–RBD interface. This suggests that the use of an unmodified V_K_ 3-20 sequence might result in an antibody with a similar if not identical binding affinity to the RBD. Thus, the light chain’s contribution to CoV11’s affinity to the RBD is largely the result of the choice of the light chain germline gene and not the somatic mutations within the gene.

### 2.5 The CDR H3 of CoV11 makes some unique interactions with the RBD to increase affinity

CDR H3 residues are not encoded by the V_H_ 3-53 germline sequence but instead arise from heavy chain joining and diversity genes. Anti-SARS-CoV-2 V_H_ 3-53 encoded antibody CDR H3s can therefore differ significantly. The V_H_ 3-53 germline sequence ends at position 94 (**Figure S2**). $Arg_{FWR\;H3}^{94}$ makes hydrogen bonds to RBD residues $Asn_{RBDwt}^{487}$ and $Tyr_{RBDwt}^{498}$ and van der Waals contacts with $Phe_{RBDwt}^{486}$ (**Figure 2B**). Only one anti-SARS-CoV-2 V_H_ 3-53 encoded neutralizing antibody with a similar binding mode to the RBD whose structure is available has a residue other than arginine at this position, BG1-22 (PDB ID 7M6F). BG1-22 is also the antibody with the longest CDR H3, 19 amino acids; the shorter side chain at position 94 may facilitate the accommodation of the longer CDR H3 by eliminating hydrogen bonds to $Asn_{RBDwt}^{487}$ or $Tyr_{RBDwt}^{498}$ (**Figure S2**). Many anti-SARS-CoV-2 V_H_ 3-53 encoded neutralizing antibodies also have a hydrophilic residue at position 97, usually Asp, Glu, Tyr, or Gln. CoV11 has $Glu_{CDR\;H3}^{97}$ . Only the longer side chain of Glu preferentially forms a hydrogen bond with $Tyr_{RBDwt}^{453}$ over $Lys_{RBDwt}^{417}$ . This may help explain CoV11’s resistance to gamma (P.1), which has a Lys-to-Thr mutation and beta (B.1.351) and omicron (B.1.1.529.1 or BA.1), which have a Lys-to-Asn mutation.

**Figure 3:**
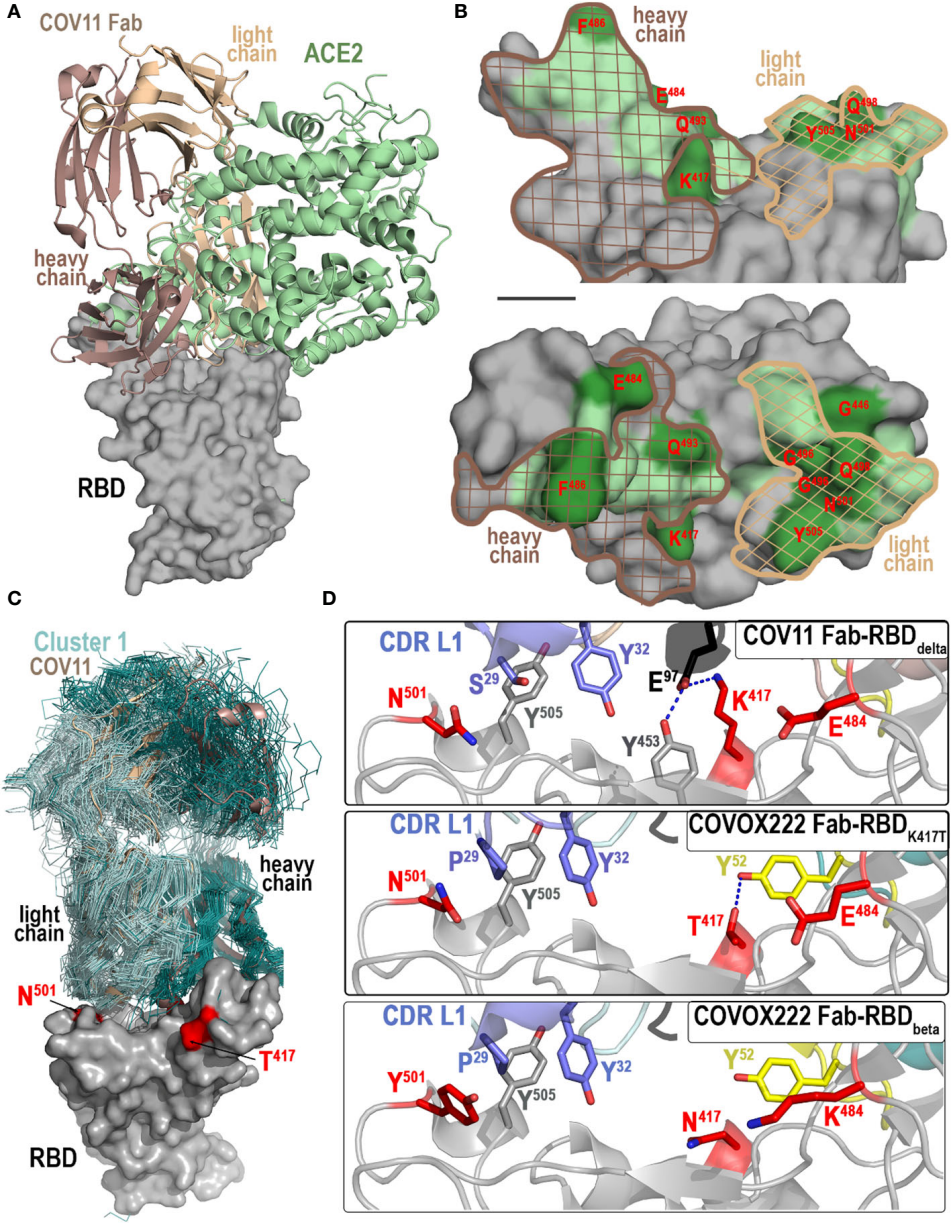
Molecular basis of CoV11’s interaction with the RBD. **(A, B)** Comparison of the binding modes of CoV11 and ACE2 to the receptor. **(A)** The structure of the ACE2-RBD_wt_ complex (PBD ID: 6VW1) was overlaid over the CoV11 Fab-RBD_wt_ complex (based on the RBD). Surface is displayed over the RBD molecule with CoV11 and ACE2 shown as cartoons. **(B)** Epitope footprint of heavy (dark brown mesh) and light (light brown mesh) chain of CoV11 is shown over the ACE2 footprint (green). RBD residues that mutate in VOCs are shown in darker green and labeled in red. **(C, D)** Recognition of RBD by CoV11 and RBD-specific Cluster 1 V_H_ 3-53 or V_H_ 3-66 germline-derived antibodies. **(C)** Available crystal structures of Cluster 1 antibodies in complex with the RBD of various SARS-CoV-2 variants (58 total structures) and the CoV11 Fab-RBD_delta_ complex were superimposed based on the RBD. Fabs of Cluster 1 V_H_ 3-53 or V_H_ 3-66 germline-derived antibodies are shown as ribbons (colored dark and light cyan for heavy and light chain, respectively) and the Fabs of CoV11 are shown as a cartoon (dark and light wheat for heavy and light chain, respectively). The RBD contact residues that mutate in the delta and beta strains are shown in red. **(D)** Close-up view of the interaction network mediated by CoV11 and COVOX222 (PDB IDs: 7NX8 and 7NXA) to RBD residues 417, 484, and 501 that mutate in VOCs. Complex interface residues are shown as sticks and H-bonds are shown as blue dashed lines.

The remaining interactions of CoV11’s CDR H3 with the RBD are mainly hydrophobic, $Leu_{CDR\;H3}^{96}$ , $Val_{CDR\;H3}^{98}$ , , and $Tyr_{CDR\;H3}^{102}$ . While many other V_H_ 3-53 encoded anti-SARS-CoV-2 neutralizing antibodies also have hydrophobic residues at position 96, some use Asp or Glu to form a salt bridge with $Lys_{RBDwt}^{417}$ . A similar situation is seen for position 98, with many V_H_ 3-53 encoded anti-SARS-CoV-2 neutralizing antibodies also encoding a hydrophobic amino acid. The exceptions are largely due to those antibodies that encode a residue that can form a hydrogen bond or salt bridge with $Lys_{RBDwt}^{417}$ . Position 99 is more universally hydrophobic. Exceptions are those of Tyr, Lys, or Ser that form hydrogen bonds or salt bridges with $G\ln_{RBDwt}^{493}$ or $Glu_{RBDwt}^{484}$ , residues that are also mutated in some VOCs (**Figures 3A, B**). Position 102 at the end of CDR H3 is almost always Tyr, Val, or Ile; however, some antibodies in this class with longer CDR H3s use other CDR H3 residues to make similar contacts with the RBD. Those that do use position 102 make equivalent contacts with the RBD using Ile, Val, or Tyr; CoV11 uses $Tyr_{CDR\;H3}^{102}$ . These CDR H3 differences influence antibody neutralization breadth with those minimizing contacts to $Lys_{RBDwt}^{417}$ , $G\ln_{RBDwt}^{493}$ , and $Glu_{RBDwt}^{484}$ , residues mutated in VOCs, having greater neutralization breadth for current VOCs. Therefore, CoV11 is among the anti-SARS-CoV-2 V_H_ 3-53 encoded antibodies with the greatest neutralization breadth for current VOCs.

### 2.6 CoV11 uses similar but not identical residues to ACE2 to bind the RBD

The CoV11 and ACE2 binding sites on the RBD largely overlap, but there are residues unique to each interface and those that are used by both are not always used in the same way (**Figures 3A, B**). These differences set a limit on which residues can be changed by the virus and not disrupt receptor binding. CoV11 makes contact with 20 residues in common with ACE2 on the RBD (residues 415, 417, 449, 453, 455, 456, 473, 475–476, 486–487, 489–490, 493, 496, 498, 500–502, and 505) and 11 residues that are not in the ACE2 interface (residues 403, 405, 416, 420–421, 457–460, 474, and 477) (**Figure 3C**). ACE2, on the other hand, only utilizes nine residues that are absent from the CoV11 epitope (residues 408, 439, 446, 484–485, 492, 499, 503, and 506).

Eleven residues in the ACE2 binding site have changed in VOCs to date (currently omicron BA.5 and XBB1.5), seven of which are also used by CoV11 (Arg^408^ to Ser [omicron BA.2 and BA.4/5], Lys^417^ to Asn [beta and omicron variants] or Thr [gamma], Gly^446^ to Ser [omicron BA.3], Glu^484^ to Lys [beta and gamma] or Ala [omicron variants], Phe^486^ to Val [omicron BA.4/5] or Pro [omicron XBB1.5], Phe^490^ to Ser [omicron XBB1.5], Gln^493^ to Arg [omicron BA.1, BA.2, BA.3, and XBB1.5], Gly^496^ to Ser [omicron BA.1], Gln^498^ to Arg [omicron variants], Asn^501^ to Tyr [alpha, beta, gamma, and omicron variants], and Tyr^505^ to His [omicron variants]). In contrast, only three of the residues that are unique to the CoV11 epitope have changed (Asp^405^ to Asn [omicron BA.2, BA.3, BA.4/5, and XBB1.5], Asn^460^ to Lys [omicron XBB1.5], and Ser^477^ to Asn [omicron variants], shown combined in **Figure 3B**).

## 4 Discussion

The first VOC mutation to occur in the RBD (Alpha, B.1.1.7), Asn501 to Tyr, increased the RBD’s affinity to ACE2 and recapitulated the enhanced infectivity seen in Alpha relative to the original SARS-CoV-2 strain (37); Tyr potentially preserves the polar interactions of Asn ( $Tyr_{ACE2}^{41}$ ), but strengthens the hydrophobic interactions with the aliphatic portion of other ACE2 interacting residues ( $Lys_{ACE2}^{353}$ and $Asp_{ACE2}^{355}$ ). Three other VOC mutations (those at Gly^446^, Phe^490^, and Gly^496^) are predicted to have little or no effect on ACE2 affinity since they are interface contacts solely made to main chain RBD atoms. Most other VOC mutations change ACE2’s affinity to the RBD. Mutations in VOCs that have appeared more recently are more disruptive of the ACE2-RBD interface, suggesting that their function may be geared more towards immune evasion. The Arg^408^-to-Ser mutation in omicron BA.2 and BA.4/5 potentially removes a hydrogen bond between the RBD and the glycan linked to $Asn_{ACE2}^{90}$ . The Lys^417^ mutation to Thr or Asn in gamma or beta and omicron VOCs respectively removes a salt bridge between Lys^417^ and $Asp_{ACE2}^{30}$ . Glu^484^ to Lys or Ala in beta or omicron VOCs removes a salt bridge between Glu^484^ and $Lys_{ACE2}^{31}$ . Phe^486^ to Val or Pro (omicron BA.4/5 or XBB1.5) changes the hydrophobic packing between Phe^486^ and $Leu_{ACE2}^{79}$ , $Met_{ACE2}^{82}$ , and $Tyr_{ACE2}^{83}$ . Gln^493^ to Arg (omicron BA.1–3 and XBB1.5) potentially removes a hydrogen bond to $Lys_{ACE2}^{31}$ but maintains one to $Glu_{ACE2}^{35}$ . Finally, Tyr^505^ to His in omicron VOCs potentially maintains a hydrogen bond to $Glu_{ACE2}^{37}$ but removes ones to $Lys_{ACE2}^{353}$ and $Arg_{ACE2}^{393}$ . The observation that some of these mutations have reverted back to wt, e.g., Gln^493^ to Arg in omicrons BA.1–3 has gone back to Gln in BA.4/5, suggests that the virus is paying a price for these changes. It also suggests that these changes are less drastic than they could be if antibody evasion was their only criteria for incorporation into the RBD. Some of these changes in the RBD may have been in response to antibodies like CoV11.

### 4.1 Changes in sequence from V_H_ 3-53 germline and the CDR H3 sequence influence neutralization breadth

While it is impossible to predict which changes from the germline sequence are the most important for increasing antibody neutralization breadth, the structure offers some insight. The first is that the heavy chain V_H_ 3-53 and V_H_ 3-66 germline sequences are essentially equivalent for this mode of binding to the RBD. The V_H_ 3-66 germline sequence differs from the V_H_ 3-53 sequence in one position, 12 (**Figure S2**). $Val_{FWR\;H1}^{12}$ in V_H_ 3-66 and $Ile_{FWR\;H1}^{12}$ in V_H_ 3-53 sit well outside of the epitope footprint. This essentially doubles the number of naïve germline sequences that can serve as starting points for RBD binding. The second is that two changes relative to the heavy chain germline sequence are able to significantly increase CoV11’s affinity to the RBD, $Thr_{FWR\;H1}^{28}$ to Ile and $Ser_{CDR\;H1}^{31}$ to Arg. Twelve other anti-SARS-CoV-2 V_H_ 3-53 or V_H_ 3-66 antibodies of this type have a similar mutation at position 28 (13 total of 58 sequences or 22%), and six others have a similar mutation at position 31 (7 total of 58 sequences or 12%) (**Figures 3D**, **S2**). Approximately 50% that do not have the $Thr_{FWR\;H1}^{28}$ -to-Ile mutation have a mutation of the adjacent $Phe_{FWR\;H1}^{27}$ to a smaller hydrophobic residue, Val, Ile, or Leu, which may imply a similar function and a common mechanism of increasing RBD affinity. In contrast, only one sequence has a change from $Ser_{CDR\;H1}^{31}$ to anything other than Arg (2%), suggesting that this is a less common means of increasing RBD affinity (14% combined). The only other CoV11 change from the heavy chain germline sequence is the conservative mutation of $Tyr_{CDR\;H2}^{58}$ to Phe, which is present in 28 (48%) of the other heavy chain germline sequences. Only two are mutated to any other residue (Asp, 3%), suggesting that this residue is important for binding the RBD even though its mutation from the germline $Tyr_{CDR\;H2}^{58}$ might not provide much benefit. A large percentage of mutations in the germline sequence occur outside of the epitope footprint. For CoV11, this only amounts to one of the four heavy chain mutations, but for some of the other examples, it can be more than half of the mutations present, which suggests that the process of affinity maturation also introduces many functionally silent mutations.

Sequences outside of the V_H_ 3-53 germline sequence also contribute to neutralization breadth. CoV11 has $Glu_{CDR\;H3}^{97}$ at position 97 (**Figure 2B**). Only the longer side chain of Glu preferentially forms a hydrogen bond with $Tyr_{RBDwt}^{453}$ over $Lys_{RBDwt}^{417}$ . $Lys_{RBDwt}^{417}$ ’s mutation to Thr or Asn in gamma, beta, and omicron RBDs supports the incorporation of this preference in the CDR H3. Only three other antibodies with structures available of this type have a similar $Glu_{CDR\;H3}^{97}$ , EH3, COVA2-04, and BG4-25. Most other antibodies of this class are less tolerant of mutations at $Lys_{RBDwt}^{417}$ . The remaining CDR H3 residues of CoV11 used to interact with the RBD are mainly hydrophobic, $Leu_{CDR\;H3}^{96}$ , $Val_{CDR\;H3}^{98}$ , $Ala_{CDR\;H3}^{99}$ , and $Tyr_{CDR\;H3}^{102}$ . While hydrogen bonds and/or salt bridges contribute to CDR H3 affinity for other antibodies of this type, they generally do so to residues that have mutated in VOCs. The preferential use of hydrophobic van der Waals interactions in the CDR H3 may be a contributing factor to neutralization breadth.

### 4.2 The choice of light chain influences neutralization breadth

CoV11’s light chain has four mutations relative to its V_K_ 3-20 germline sequence. However, none of these residues is directly involved in the RBD binding interface; only the main chain atoms of residue 28 are used in the CoV11 Fab-RBD interface, making the $Val_{CDR\;L1}^{28}$ -to-Ile change of little impact with respect to binding affinity. Also, unlike the case for the heavy chain, the V_K_ 3-20 germline sequence contains the full light chain epitope footprint. This implies that a light chain with an unmutated V_K_ 3-20 germline sequence would serve CoV11 as well as the one it has. Supporting this observation, of the 15 antibodies with V_K_ 3-20 germline sequences paired to a V_H_ 3-53 or V_H_ 3-66 heavy chain, 5 (33%) have unmutated V_K_ 3-20 germline sequences.

There are 58 structures of complexes of V_H_ 3-53 or V_H_ 3-66 heavy chain antibodies in complex with the RBD that bind like CoV11 (**Figure 3D** and **S3**). Fifteen of these are paired to a V_K_ 3-20 germline-derived light chain. The most commonly paired light chain gene for these antibodies is V_K_ 1-9 (17 antibodies), followed by V_K_ 3-20 (15), V_K_ 1D-33 (9), and V_K_ 1-39 (6). There are four other kappa light chain germline gene examples with only one example of each, V_K_ 1-12, V_K_ 1-5, V_K_ 1S8, and V_K_ 4-1. Examples with lambda light chain germline genes are rarer with only seven total in the dataset. Two have two examples each, V_L_ 1-40 and V_L_ 3-21. The remaining genes have only one example each, V_L_ 1-9, V_L_ 2-8, and V_L_ 1-44. While these germline genes represent a variety of sequences, the portions that interact with the RBD are surprisingly similar.

As shown in **Figure 3D**, CoV11 uses the side chain of $Ser_{CDR\;L1}^{27A}$ to form a hydrogen bond with the main chain of $Gly_{RBDwt}^{502}$ . All other antibodies using the V_K_ 3-20 germline sequence share this feature with two exceptions that have deletions of residue 27A (**Figure S3**). The two V_K_ 3-20 germline exceptions and all but one other antibody with kappa light chains make a similar hydrogen bond when possible (approximately 40% are $Gly_{CDR\;L1}^{28}$ and cannot) with $Ser_{CDR\;L1}^{28}$ or $Asp_{CDR\;L1}^{28}$ . Lambda light chains also tend to shift this interaction to position 28 even though most have longer CDR L1s. $Ser_{CDR\;L1}^{29}$ in CoV11 and most other V_K_ 3-20 germline sequence light chains pack against $Tyr_{RBDwt}^{505}$ . However two V_K_ 3-20 germline sequences have mutated $Ser_{CDR\;L1}^{29}$ to ${\mathrm{Pro}}_{CDR\;L1}^{29}$ , BG4-25 and COVOX222 (**Figure 3D** and **S3**). Pro at position 29 also packs well against the $Tyr_{RBDalpha}^{501}$ mutation seen in every VOC since alpha (**Figure 2D**), which may indicate that these antibodies were elicited against alpha and not wt RBD. Most other kappa light chain antibodies use $Ile_{CDR\;L1}^{29}$ to pack against $Tyr_{RBDwt}^{505}$ , but, possibly because of a shorter CDR L1, form stronger hydrogen bonds between the often common Ser at position 30 and $Asn_{RBDwt}^{501}$ , making the $Tyr_{RBDalpha}^{501}$ mutation more disruptive. Tyr at position 32 near the end of CDR L1, or Phe or Trp in a few cases, is then used to pack against $Tyr_{RBDwt}^{505}$ , ending CDR L1’s interaction with the RBD.

CDR L2 makes little or no contribution to the interface for almost all of these paired light chains, independent of type (**Figures 2A, C**, **Table S1**). CDR L3’s interaction with the RBD with the exception of lambda and V_K_ 1-39 light chains usually begins with a hydrogen bond from residue 90 to $Tyr_{RBDwt}^{505}$ using Gln with the occasional His, Glu, or Arg. $G\ln_{CDR\;L3}^{90}$ and $Glu_{CDR\;L3}^{90}$ have the advantage in that they can still form a hydrogen bond to $His_{RBDomicron}^{505}$ . The exceptions to this rule are mostly the lambda and V_K_ 1-39 light chains, which shift their interaction with the RBD further along their CDR L3s. The remaining contacts in CoV11 are mediated by $Gly_{CDR\;L3}^{92}$ and $Ser_{CDR\;L3}^{93}$ . With the exception of COVOX222, which has $Asp_{CDR\;L3}^{92}$ and $Thr_{CDR\;L3}^{93}$ , these residues are invariant in V_K_ 3-20 light chains. The residues and sequences used by other light chains are more diverse with fewer common features. Most kappa light chains other than those derived from the V_K_ 3-20 germline gene seem to focus part of their CDR L3 to forming a hydrogen bond or salt bridge with $Lys_{RBDwt}^{417}$ , making them more susceptible to a mutation there. This is less common for the lambda light chains.

## 5 Conclusions

In conclusion, the SARS-CoV-2 spike protein has changed over the course of the pandemic to increase infectivity and to evade immune pressure. This has led to mutations in the spike protein that have diminished the neutralization potency of a large number of antibodies elicited either by natural infection or by vaccination. Some of the most potent of these antibodies directly compete with ACE2 in binding the RBD (38–40). The low level of somatic mutation in one group of these antibodies enabled their early identification in B-cell sequences of infected individuals (11). This group of antibodies derived from the heavy chain V_H_ 3-53 or V_H_ 3-66 germline sequences with generally short CDR H3s were later found to almost directly overlap the ACE2 binding site on the RBD, providing a clear explanation for their mode of neutralization of the virus (11, 13–19).

Mutations in the virus in viral variants have affected antibodies of this class as they have done to all SARS-CoV-2 neutralizing antibodies. The first widely spread mutation that out-competed the original strain, $Asn_{RBDwt}^{501}$ to Tyr in the alpha variant (37), mostly affected the light chain of this group of antibodies and many were able to accommodate this change with little change to receptor affinity. Other more recent mutations in VOCs that greatly affected other antibody classes (22), e.g., the $Glu_{RBDwt}^{484}$ to Lys or Ala in the beta, gamma, and omicron variants, have also had little effect on receptor affinity for this group of antibodies. One of the most detrimental for this type of antibodies has been changes from $Lys_{RBDwt}^{417}$ to Thr or Asn in the gamma, beta, and omicron variants. CoV11 is an example of an antibody of this class that depends less strongly on a salt bridge or hydrogen bond to $Lys_{RBDwt}^{417}$ due to unique characteristics of its heavy chain CDR H3. While CoV11’s affinity to RBDs containing mutations of $Lys_{RBDwt}^{417}$ is lower, the fact that both Fabs of a CoV11 IgG can interact with a single spike protein partially counteracts this loss in affinity in neutralization.

The majority of residues that make up CoV11’s epitope footprint are shared with ACE2 in binding the RBD. This ensures that these residues cannot change without also affecting the RBD’s affinity to ACE2 and places a constraint on mutations that can occur in VOCs. CoV11 can still neutralize omicron BA.1 with only a slightly higher IC_50_ than it does beta, the variant with the next highest IC_50_ in spite of the fact that omicron BA.1 incorporates many more mutations to the RBD. Small changes to CoV11, e.g., the $Ser_{CDR\;L1}^{29}$ -to-Pro change used by COVOX222’s light chain, could overcome some of these losses in affinity to VOCs. Additional changes in the most recent VOCs such as the Asn^460^-to-Lys change in omicron BQ.1.1 or the Phe^486^-to-Pro, Gln^493^-to-Arg, and Asn^460^-to-Lys changes in omicron XBB.1.5 are enough to reduce CoV11’s affinity to the point where it no longer effectively neutralizes the virus (**Figures S2** and **S3**). SPR and BLI data indicate that much of this loss in affinity is due to increases in the off-rate (*k*_d_) rather than decreases in the on-rate (*k*_a_) of CoV11, which suggests that small changes in CoV11’s sequence could allow CoV11 to compensate. For example, the Phe^486^-to-Pro change in XBB.1.5 could be ameliorated with a Val^2^-to-Phe change in CoV11’s heavy chain; Val^2^ is one of CoV11’s main contact residues with Phe^486^ and mutating it to Phe restores the contact in XBB.1.5. Other changes to restore binding and increase neutralization breadth in Omicron variants are less straightforward to predict due to the sheer number of amino acid changes possible at each position. CoV11 was elicited early in the course of the pandemic. Continued rounds of somatic mutation and selection to VOCs could easily generate a more potent and broadly neutralizing version that is more adapted to current VOCs.

The high prevalence of V_H_ 3-53 and V_H_ 3-66 germline sequences in the naïve antibody pool has probably helped in the immune response to SARS-CoV-2 in the general population. However, antibody selection and maturation is a stochastic process and not all individuals will utilize these germline sequences in response to SARS-CoV-2. There are many equally good responses to the antigen in its initial incarnation. It is only during the course of the pandemic that some responses have been shown to be better at neutralizing multiple strains than others. Antibodies derived from the V_H_ 3-53 and V_H_ 3-66 germline sequences have fared relatively well to date mainly because they share a majority of residues in common with ACE2 in their epitope footprint. While it is not possible to predict how the virus will continue to change in the future, CoV11 represents a good starting point for the design of a broad neutralizing response to SARS-CoV-2. With some minor changes, it could be made to be an even more broad and potent neutralizing therapeutic antibody for current VOCs and possibly also for the next generation of VOCs.

## Data availability statement

The datasets presented in this study can be found in online repositories. The names of the repository/repositories and accession number(s) can be found here: 7S4S, 7URQ, and 7URS (Protein data bank).

## Author contributions

WT, MS, and MP conceptualized this study and designed the experiments. WT, YC, RM, EP, and SG produced, purified, and characterized the proteins. WT and MP solved and analyzed the crystal structures of complexes. YC performed SPR kinetics. YC and LS generated PsVs and performed neutralization assays. MB performed BLI kinetics, SD performed neutralization of omicron variants. MC generated the BQ.1.1 and XBB.1.5 spike-expressing plasmids. ZT and MS isolated the CoV11 antibody. WT, MS, AF, and MP analyzed data. MP, FG, AF, and MS funded the work. WT and MP wrote the manuscript and every author has read, edited, and approved the final manuscript. All authors contributed to the article and approved the submitted version.

## Supplementary material

Supplementary Figure 1 Representative SPR kinetic measurements of RBD antigens binding to immobilized CoV11. All measurement were performed with ~80-200 RU CoV11 IgG immobilized on a Protein A chip. Flow RBD antigens were injected at the indicated concentrations. The background-corrected sensorgrams (colored) were fitted with a 1:1 Langmuir model (grey). Kinetic constants are summarized in **Table S2**.

Supplementary Figure 2 Representative BLI kinetic measurements of RBD antigens binding to immobilized CoV11. All measurement were performed with CoV11 IgG immobilized on a Protein A sensor tip. Sensor tips were exposed to RBD antigens at the indicated concentrations. The background-corrected sensorgrams (colored) were fitted with a 1:1 Langmuir model (grey). Kinetic constants are summarized in **Table S3**.

Supplementary Figure 3 Neutralization activity of COV11 IgG against SARS-CoV-2 D614G (positive control) or Omicrons BQ.1.1 and XBB.1.5. Dose response neutralization curves of SARS-CoV-2 pseudotyped lentivirus with SAR2-CoV-2 S variants. hACE2 expressing 293T cells were infected with different variants of SARS-CoV-2 PsV or VSVG PsV (negative control) in the presence of CoV11 IgG. Infectivity was quantified by the cellular luciferase signal 48 hours post infection. Relative infectivity was normalized by the luciferase signal in infected cells without intervention (PBS saline).

Supplementary Figure 4 Heavy chain sequence alignment of V_H_ 3-53 or V_H_ 3-66 IgGs that bind the RBD like CoV11. Antibodies are labeled by PDB ID underscore antibody name and residues are numbered using the Kabat antibody number scheme. Contact residues defined by a 5 Å cutoff are marked above the sequence with (+) for side chain and (-) for main chain to indicate the type of contact. Contact types are colored as follows: hydrophilic (blue), hydrophobic (green) and both (black). Residues that differ from the V_H_ 3-53 or V_H_ 3-66 germline sequence colored red. The one residue difference in the V_H_ 3-66 germline sequence relative to the V_H_ 3-53 germline sequence is colored blue. CDRs are colored as in .

Supplementary Figure 5 Light chain sequence alignment of IgGs that bind the RBD like CoV11. Antibodies are labeled by PDB ID underscore antibody name and residues are numbered using the Kabat antibody number scheme. Contact residues defined by a 5 Å cutoff are marked above the sequence with (+) for side chain and (-) for main chain to indicate the type of contact. Contact types are colored as follows: hydrophilic (blue), hydrophobic (green) and both (black). Residues that differ from the V_K_ 3-20 germline sequence colored red. Only the first 15 sequences utilize the V_K_ 3-20 germline sequence. The last five use lambda germline sequences and the remaining sequences use kappa germline sequences other than V_K_ 3-20. CDRs are colored as in .

Supplementary Table 1 Details of the CoV11 Fab-RBD_wt_, CoV11 Fab-RDB_delta_, C098 Fab-RBD_wt_ (germline V_H_ 3-53 and V_K_ 3-20), and ACE2-RBD_wt_ interfaces as calculated by the EBI PISA server (http://www.ebi.ac.uk/msd-srv/prot_int/cgi-bin/piserver).

Supplementary Table 2 Surface Plasmon Resonance (SPR) kinetic constants of CoV11 binding to the RBD of SARS-CoV-2 wt and selected VOCs. CoV11 IgG was immobilized on Protein A chip and the RBDs of various SARS-CoV-2 strains were injected as flow analytes. The dissociation constants (K_D_), association constants (k_a_) and dissociation constants (k_d_) are as shown. K_D_ values were determined using a 1:1 Langmuir model. Experimental binding curves are shown in **Figure S1**.

Supplementary Table 3 Bio-Layer Interferometry (BLI) kinetic constants of CoV11 binding to the RBD of SARS-CoV-2 wt and selected VOCs. CoV11 IgG was immobilized on a Protein A sensor tip and then placed into solutions containing the RBDs of various SARS-CoV-2 strains at different concentrations. The dissociation constants (K_D_), association constants (k_a_) and dissociation constants (k_d_) are as shown. K_D_ values were determined using a 1:1 Langmuir model. Values are the average of two or three experiments with standard deviations as shown. N.D., not detected. Experimental binding curves are shown in **Figure S2**.
