# Supplementary material for: The molecular basis of the neutralization breadth of the RBD-specific antibody CoV11
Source: Front Immunol. 2023 Jun 2;14:1178355. doi: 10.3389/fimmu.2023.1178355 (PMC10272436; doi:10.3389/fimmu.2023.1178355)
Supplement: Supplementary Figure 1 — Representative SPR kinetic measurements of RBD antigens binding to immobilized CoV11. All measurement were performed with ~80-200 RU CoV11 IgG immobilized on a Protein A chip. Flow RBD antigens were injected at the indicated concentrations. The background-corrected sensorgrams (colored) were fitted with a 1:1 Langmuir model (grey). Kinetic constants are summarized in Table S2 . [file DataSheet_1.docx]

Supplementary Material

1. **Supplementary Figures and Tables**
   1. **Supplementary Tables**

**Table S1. Details of the CoV11 Fab-RBD_wt_, CoV11 Fab-RDB_delta_, C098 Fab-RBD_wt_ (germline V_H_ 3-53 and V_K_ 3-20), and ACE2-RBD_wt_ interfaces** as calculated by the EBI PISA server (<http://www.ebi.ac.uk/msd-srv/prot_int/cgi-bin/piserver>).

|  | | CoV11 Fab-RBD_wt_  (7S4S) | CoV11 Fab-RDB_delta_  (7URQ) | C098 Fab- RBD_wt_  (7N3I) | ACE2- RBD_wt_  (6VW1) |
| --- | --- | --- | --- | --- | --- |
| **Buried Surface Area, Å²** | **RBD total** | **1130** | **1121** | **1130** | **930** |
|  | RBD ridge | 213 | 219 | 186 | 247 |
|  | RBM | 875 | 861 | 889 | 920 |
|  | **Heavy chain total** | **861** | **867** | **753** | — |
|  | FWR | 146 | 170 | 141 | — |
|  | CDR H1 | 231 | 213 | 165 | — |
|  | CDR H2 | 246 | 251 | 239 | — |
|  | CDR H3 | 238 | 233 | 208 | — |
|  | **Light chain total** | **311** | **297** | **388** | — |
|  | FWR | 17 | 6 | 40 | — |
|  | CDR L1 | 233 | 229 | 282 | — |
|  | CDR L2 | 3 | 0 | 0 | — |
|  | CDR L3 | 58 | 62 | 66 | — |
|  | **Heavy and light chain total** | **1172** | **1164** | **1141** | — |
|  | **ACE2 total** | — | — | — | **838** |

**Table S2. Surface Plasmon Resonance (SPR) kinetic constants of CoV11 binding to the RBD of SARS-CoV-2 wt and selected VOCs.** CoV11 IgG was immobilized on Protein A chip and the RBDs of various SARS-CoV-2 strains were injected as flow analytes. The dissociation constants (K_D_), association constants (k_a_) and dissociation constants (k_d_) are as shown. K_D_ values were determined using a 1:1 Langmuir model. Experimental binding curves are shown in **Figure S1**.

| **Immobilized ligand** | **Flow analyte** | **Fitting mode** | ***k*_a_ (M^-1^ s^-1^)** | ***k*_d_ (s^-1^)** | **K_D_ (nM)** | **chi^2^ value** | **K_D_ fold increase as compared to RBD_wt_** |
| --- | --- | --- | --- | --- | --- | --- | --- |
| CoV11  IgG | RBD_wt_ | 1:1 | 1.2 x 10^5^ | 2.3 x 10^-4^ | 1.9 | 1. 57 | 1 |
|  | RBD_alpha_ | 1:1 | 1.8 x 10^4^ | 2.3 x 10^-3^ | 3. 7 | 2.76 | 1.9 |
|  | RBD_beta_ | 1:1 | 6.7 x 10^5^ | 6.9 x 10^-3^ | 10.4 | 1.29 | 5.5 |
|  | RBD_epsilon_ | 1:1 | 2.1 x 10^5^ | 9.1 x 10^-4^ | 4.4 | 1.81 | 2.3 |
|  | RBD_iota_ | 1:1 | 2.5 x 10^5^ | 1.2 x 10^-3^ | 4.9 | 4.59 | 2.6 |
|  | RBD_delta_ | 1:1 | 2.1 x 10^5^ | 7.3 x 10^-4^ | 3.5 | 1.89 | 1.8 |
|  | RBD_omicron BA.2_ | 1:1 | 2.6 x 10^5^ | 6.3 x 10^-3^ | 24.4 | 3.03 | 12.8 |

**Table S3.** **Bio-Layer Interferometry (BLI) kinetic constants of CoV11 binding to the RBD of SARS-CoV-2 wt and selected VOCs.** CoV11 IgG was immobilized on a Protein A sensor tip and then placed into solutions containing the RBDs of various SARS-CoV-2 strains at different concentrations. The dissociation constants (K_D_), association constants (k_a_) and dissociation constants (k_d_) are as shown. K_D_ values were determined using a 1:1 Langmuir model. Values are the average of two or three experiments with standard deviations as shown. N.D., not detected. Experimental binding curves are shown in **Figure S2**.

| **Immobilized ligand** | **Flow analyte** | ***k*_a_ (M^-1^ s^-1^)** | ***k*_d_ (s^-1^)** | **K_D_ (nM)** | **K_D_ fold increase as compared to RBD_wt_** |
| --- | --- | --- | --- | --- | --- |
| CoV11  IgG | RBD_wt_ | 6.9 x 10^4^ ± 4.0 x 10^3^ | 1.5 x 10^-4^ ± 2.9 x 10^-5^ | 2.1 ± 0.4 | 1 |
|  | RBD_alpha_ | 7.2 x 10^4^ ± 3.2 x 10^3^ | 1.4 x 10^-4^ ± 2.1 x 10^-5^ | 1.9 ± 0.2 | 0.9 |
|  | RBD_beta_ | 6.9 x 10^4^ ± 7.8 x 10^3^ | 1.4 x 10^-2^ ± 4.0 x 10^-3^ | 202 ± 81 | 96 |
|  | RBD_epsilon_ | 7.6 x 10^4^ ± 5.6 x 10^3^ | 1.4 x 10^-4^ ± 9.6 x 10^-5^ | 1.9 ± 1.9 | 0.9 |
|  | RBD_iota_ | 8.5 x 10^4^ ± 3.9 x 10^3^ | 2.8 x 10^-4^ ± 7.2 x 10^-6^ | 3.3 ± 0.2 | 1.6 |
|  | RBD_delta_ | 8.1 x 10^4^ ± 7.1 x 10^3^ | 1.8 x 10^-4^ ± 2.9 x 10^-5^ | 2.2 ± 0.5 | 1.04 |
|  | RBD_omicron BA.2_ | 7.5 x 10^4^ ± 7.4 x 10^3^ | 7.7 x 10^-3^ ± 4.0 x 10^-3^ | 105 ± 26 | 50 |
|  | RBD_omicron BQ.1.1_ | N.D. | N.D. | N.D. | – |
|  | RBD_omicron XBB.1.5_ | N.D. | N.D. | N.D. | – |

## Supplementary Figures

**
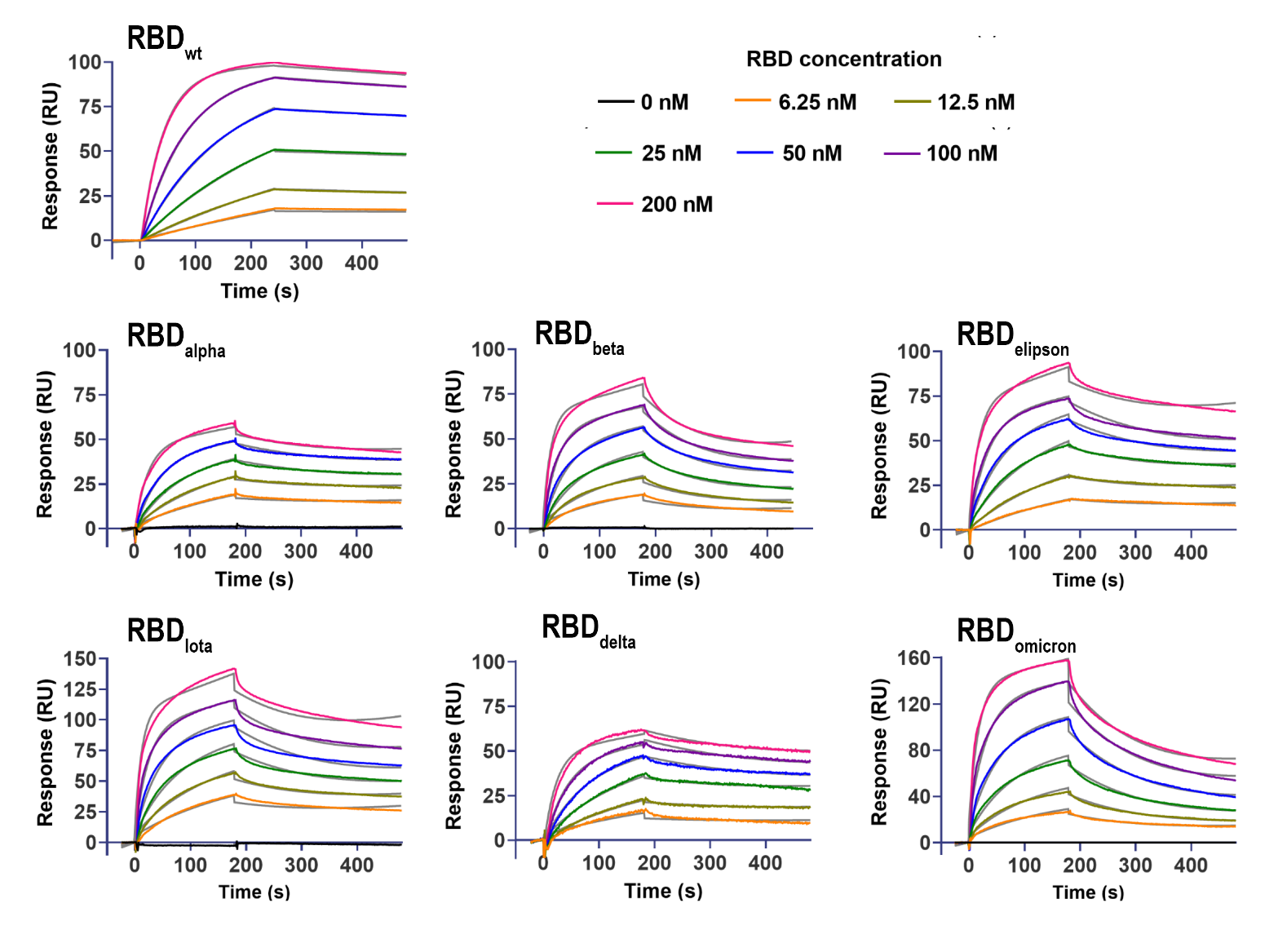
Figure S1. Representative SPR kinetic measurements of RBD antigens binding to immobilized CoV11.** All measurement were performed with ~80-200 RU CoV11 IgG immobilized on a Protein A chip. Flow RBD antigens were injected at the indicated concentrations. The background-corrected sensorgrams (colored) were fitted with a 1:1 Langmuir model (grey). Kinetic constants are summarized in **Table 2**.

**
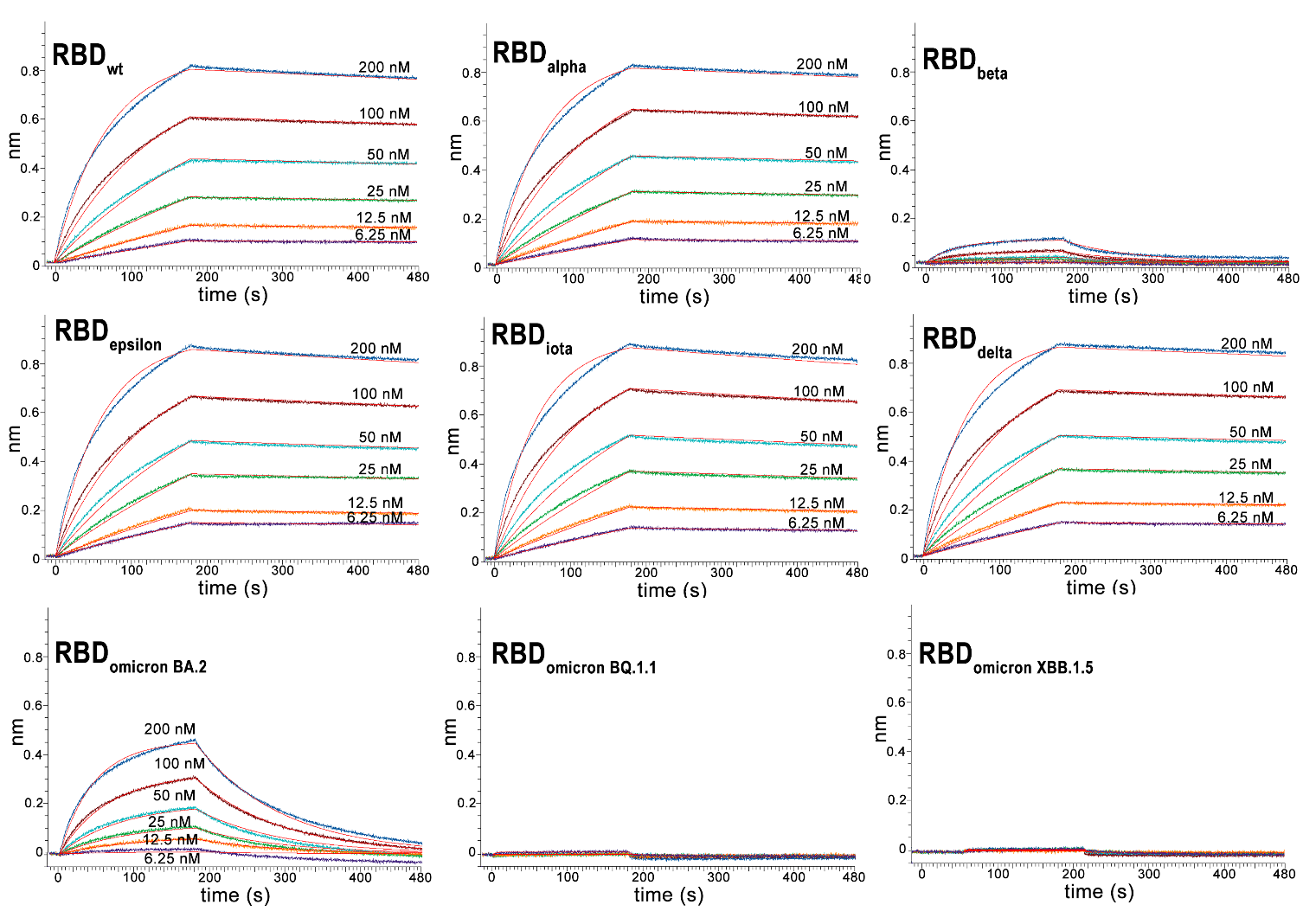
**

**S2. Representative BLI kinetic measurements of RBD antigens binding to immobilized CoV11.** All measurement were performed with CoV11 IgG immobilized on a Protein A sensor tip. Sensor tips were then exposed to RBD antigens at the indicated concentrations. The background-corrected sensorgrams (colored) were fitted with a 1:1 Langmuir model (grey). Kinetic constants are summarized in **Table 3**.


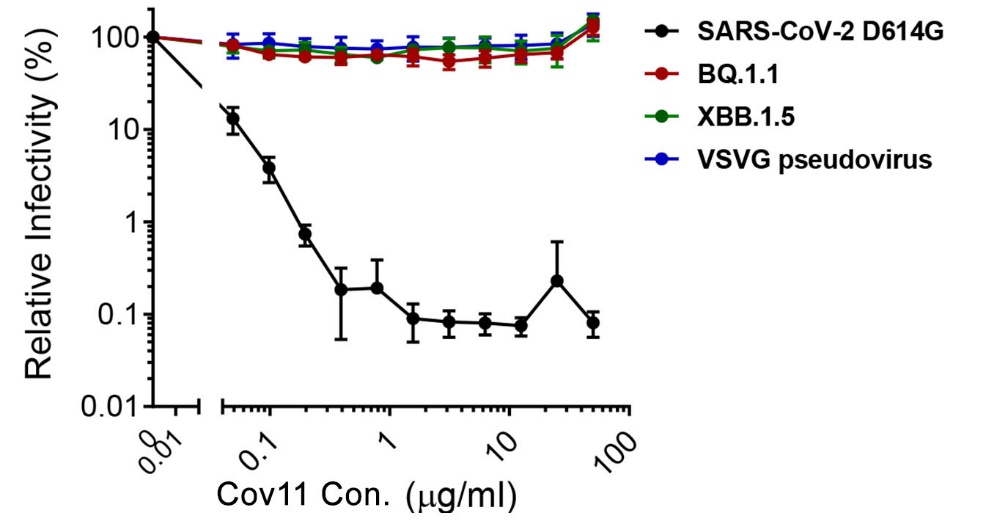


**S3. Neutralization activity of COV11 IgG against SARS-CoV-2 D614G (positive control) or Omicrons BQ.1.1 and XBB.1.5**. Dose response neutralization curves of SARS-CoV-2 pseudotyped lentivirus with SAR2-CoV-2 S variants. hACE2 expressing 293T cells were infected with different variants of SARS-CoV-2 PsV or VSVG PsV (negative control) in the presence of CoV11 IgG. Infectivity was quantified by the cellular luciferase signal 48 hours post infection. Relative infectivity was normalized by the luciferase signal in infected cells without intervention (PBS saline).


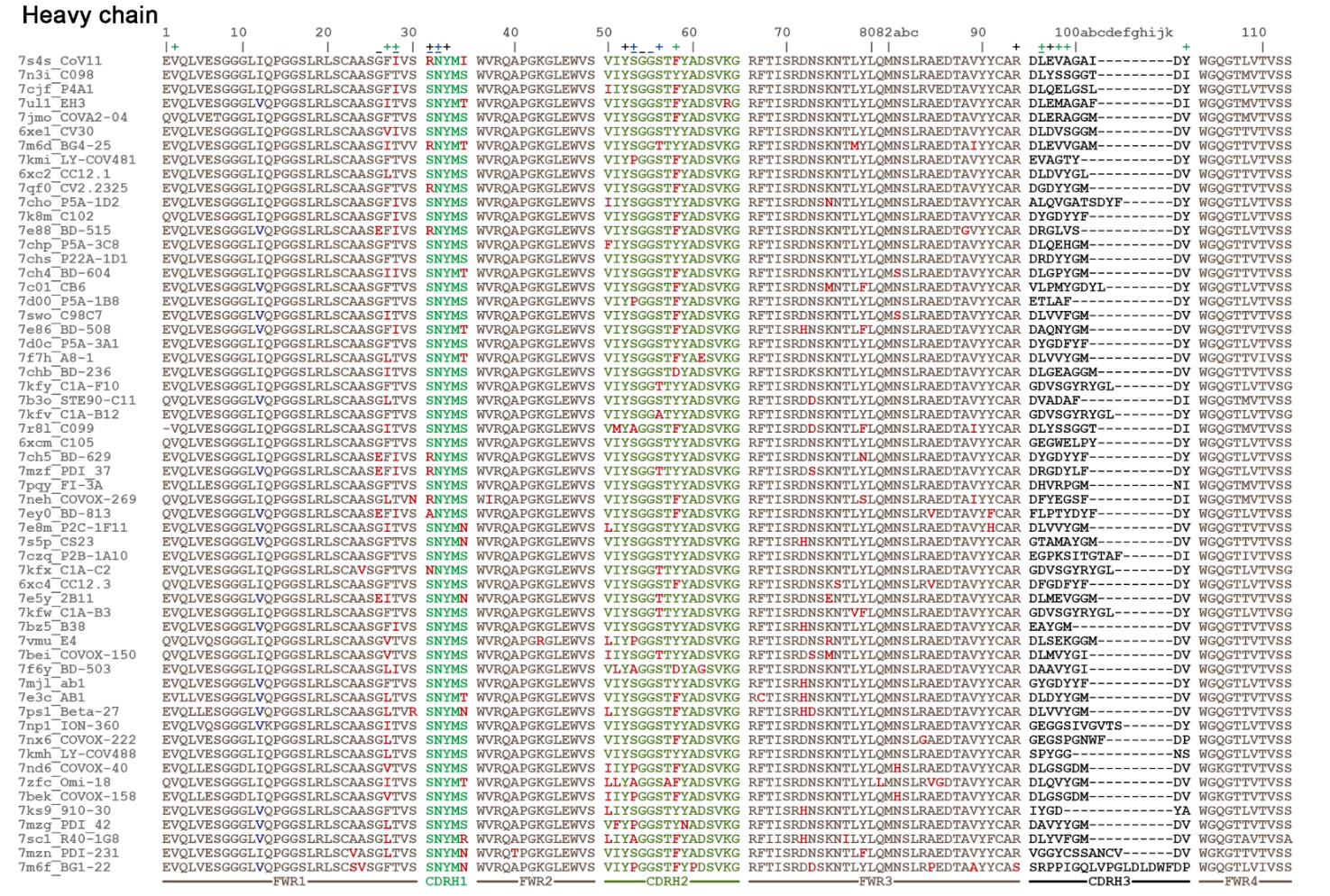


**Figure S4. Heavy chain sequence alignment of V_H_ 3-53 or V_H_ 3-66 IgGs that bind the RBD like CoV11.** Antibodies are labeled by PDB ID underscore antibody name and residues are numbered using the Kabat antibody number scheme. Contact residues defined by a 5 Å cutoff are marked above the sequence with (+) for side chain and (-) for main chain to indicate the type of contact. Contact types are colored as follows: hydrophilic (blue), hydrophobic (green) and both (black). Residues that differ from the V_H_ 3-53 or V_H_ 3-66 germline sequence colored red. The one residue difference in the V_H_ 3-66 germline sequence relative to the V_H_ 3-53 germline sequence is colored blue. CDRs are colored as in **Figure 1**.


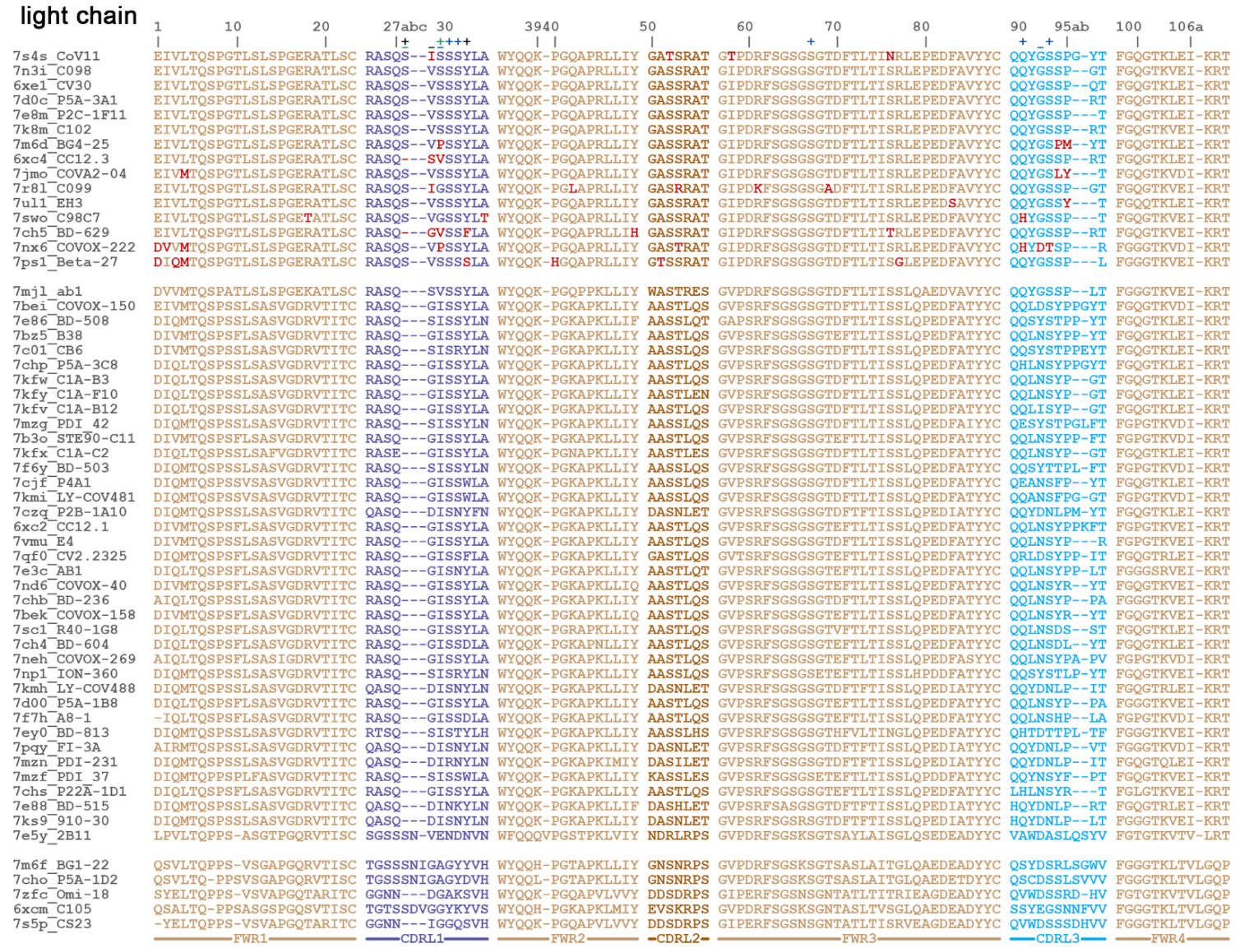


**Figure S5. Light chain sequence alignment of IgGs that bind the RBD like CoV11.** Antibodies are labeled by PDB ID underscore antibody name and residues are numbered using the Kabat antibody number scheme. Contact residues defined by a 5 Å cutoff are marked above the sequence with (+) for side chain and (-) for main chain to indicate the type of contact. Contact types are colored as follows: hydrophilic (blue), hydrophobic (green) and both (black). Residues that differ from the V_K_ 3-20 germline sequence colored red. Only the first 15 sequences utilize the V_K_ 3-20 germline sequence. The last five use lambda germline sequences and the remaining sequences use kappa germline sequences other than V_K_ 3-20. CDRs are colored as in **Figure 1.**
